# Chinese herbal injections versus intrapleural cisplatin for lung cancer patients with malignant pleural effusion: A Bayesian network meta-analysis of randomized controlled trials

**DOI:** 10.3389/fonc.2022.942941

**Published:** 2022-09-20

**Authors:** Yi-Fang Xu, Yun-Ru Chen, Fan-Long Bu, Yu-Bei Huang, Yu-Xin Sun, Cheng-Yin Li, Jodi Sellick, Jian-Ping Liu, Dan-Mei Qin, Zhao-Lan Liu

**Affiliations:** ^1^ Department of Oncology, Hubei Provincial Hospital of Traditional Chinese Medicine, Wuhan, China; ^2^ Centre for Evidence-based Chinese Medicine, School of Traditional Chinese Medicine, Beijing University of Chinese Medicine, Beijing, China; ^3^ Beijing Children’s Hospital, Capital Medical University, National Center for Children’s Health, Beijing, China; ^4^ Department of Epidemiology and Biostatistics, National Clinical Research Center of Cancer, Key Laboratory of Cancer Prevention and Therapy of Tianjin, Tianjin Medical University Cancer Institute and Hospital, Tianjin, China; ^5^ Chinese Medicine Centre, Western Sydney University, Campbelltown, NSW, Australia

**Keywords:** malignant pleural effusion (MPE), lung cancer, Chinese herbal injections, cisplatin, network meta-analysis

## Abstract

**Background:**

Malignant pleural effusion (MPE) is a common complication in patients with advanced lung cancer that can severely compromise the quality of life and limit life expectancy. Randomized controlled trials (RCTs) have shown that Chinese herbal injections (CHIs) may be beneficial in improving quality of life. This network meta-analysis (NMA) aims to explore several CHIs used for lung cancer patients with MPE.

**Methods:**

Seven databases were systematically searched for eligible RCTs from inception to November 2021. The primary outcome was the clinical effective rate. Secondary outcomes were the improvement rate of Karnofsky performance status (KPS) score and incidence of adverse events (AEs). The Cochrane risk of bias 2 tool was used to assess the quality of included studies. Data analysis was performed using STATA 16.0 and R software 4.1.0. Both pairwise meta-analysis and Bayesian NMA were conducted. Competing interventions were ranked using the surface under the cumulative ranking (SUCRA) probabilities. Evidence grading was evaluated using the Confidence in Network Meta-Analysis online software (https://cinema.ispm.unibe.ch/).

**Results:**

A total of 44 studies involving 2,573 patients were included. The combined Huachansu injection (HCS) with intrapleural cisplatin (cis-diamminedichloro-platinum, DDP) had the highest probability of improving the clinical effective rate (SUCRA, 84.33%). The Kangai injection (KA) combined with DDP had the most improvement rate of KPS score (SUCRA, 80.82%), while the Fufangkushen injection (FFKS) alone was more likely to reduce AEs including gastrointestinal reactions (SUCRA, 89.92%), leukopenia (SUCRA, 91.85%), and chest pain (SUCRA, 98.17%). FFKS combined with DDP ranked the best in reducing the incidence of fever (SUCRA, 75.45%).

**Conclusions:**

Our NMA showed that CHIs alone or combined with DDP could improve clinical effectiveness and quality of life and reduce AEs, compared to DDP alone. HSC and KA, combined with DDP, may be the most effective considering clinical effective rate and improvement of KPS score, respectively. FFKS, either used alone or in combination therapy with DDP, may be the best in reducing AEs. However, high-quality RCTs with larger sample sizes are needed to further support the evidence.

**Systematic review registration:**

PROSPERO https://www.crd.york.ac.uk/prospero/, identifier CRD42021285275.

## 1 Introduction

With an estimated crude death rate of 23% (per 100,000), lung cancer was the leading cause of cancer-related death worldwide in 2020, resulting in 1.79 million deaths (1). Throughout the disease progression, approximately 40% of patients develop pleural effusions (2). Malignant pleural effusion (MPE) usually signifies advanced-stage disease or metastasis, which is a criterion for stage IV, M1a in the TNM staging system (3), with an average survival of 4 to 7 months (2). Patients may be asymptomatic at presentation but eventually develop debilitating symptoms of dyspnea, chest pain, and cough, which severely compromise their quality of life (4).

With no cure for MPE, the main goal of current management has remained predominantly palliative to alleviate symptoms and improve quality of life (5, 6). Many treatment options include chest drainage alone or with the instillation of a pleurodesis agent, semi-permanent indwelling pleural catheter, and intracavitary chemotherapy (7). For patients with poor performance status that cannot tolerate systemic chemotherapy, intrapleural chemotherapy has been proven to be a safe and effective alternative to locally control the effusion in addition to treating the underlying malignancy (8). The most used pleural injection drug is cisplatin (cis-diamminedichloro-platinum, DDP) which can kill tumor cells and reduce the generation of pleural effusion. However, the therapeutic effect of DDP is not sufficient if used alone. Furthermore, its toxic adverse effects also need to be considered (9). Complementary and alternative treatment modalities have also been critical in cancer management. Traditional Chinese medicine (TCM) has been widely used in contemporary Chinese medical practice as an adjuvant to chemotherapy, radiotherapy, targeted therapy, and immunotherapy (10). With a number of pharmacological studies demonstrating their antitumor effects, accumulating research evidence has indicated that many medicinal plants could be used alone or in combination with commonly used chemotherapy drugs for patients with MPE, as they can increase efficiency and reduce adverse reactions (11, 12). Various kinds of Chinese herbal injections (CHIs) have been developed in recent years, containing substances extracted from single materials or compound formulas of TCM (13). Due to their extensive biological activity and low toxicity in animal studies, these drugs have been used as therapeutic options for MPE (14). Numerous randomized controlled trials (RCTs) have reported advantageous results for synergy and attenuation when CHIs have been used as adjuvant or alternative treatments when compared to DDP for lung cancer patients with MPE. While there is a diverse range of CHIs, there is insufficient evidence available to determine their effectiveness. Our study aims to conduct a systematic review and network meta-analysis (NMA) on the estimated relative effects of multiple CHIs as an adjuvant for intrapleural cisplatin (DDP) in lung cancer patients with MPE.

## 2 Methods

Our protocol has been registered with the International Prospective Register of Systematic Reviews (PROSPERO) (registration number CRD42021285275). The full review was reported in accordance with the Preferred Reporting Items for Systematic Reviews and Meta-Analyses (PRISMA) extension statement for NMA (15). The PRISMA checklist is provided in **Supplementary File S1**.

### 2.1 Search strategy

The following seven databases were searched from inception to November 2021: MEDLINE (*via* PubMed), the Cochrane Central Register of Controlled Trials (CENTRAL), EMBASE (*via* OVID), China National Knowledge Infrastructure (CNKI), WanFang Database, Chinese Scientific Journals Database (VIP), and Chinese Biomedical Literature database (SinoMed). Literature was searched using the combination of medical subject headings (MeSH), free-text words, and publication types. Only Chinese and English articles were retrieved. Reference lists of relevant systematic reviews and meta-analysis identified through screening were also checked manually. Full details of the search strategies used for each database are provided in **Supplementary File S2**.

### 2.2 Eligibility criteria

#### 2.2.1 Types of studies

Only RCTs reported in English and Chinese were included. Clinical trials described to be randomly allocated were all considered eligible, but studies with a considerable high risk of bias in the generation of the randomization sequence, for example, by date of admissions, were excluded.

#### 2.2.2 Types of patients

Adult patients over the age of 18 and diagnosed with MPE caused by lung cancer (of any type and stage), confirmed by histological or cytological findings, were included. There were no restrictions on patient gender, race, and histological types of lung cancer.

#### 2.2.3 Types of interventions

Studies that compared CHIs combined with or without DDP by intrapleural perfusion to intrapleural DDP alone were included. The following 10 CHIs, categorized as antitumor agents within the inventory of Chinese patent drugs authorized by the National Healthcare Security Administration (NHSA) of the People’s Republic of China (http://www.nhsa.gov.cn/), were considered eligible: Aidi injection (AD), Huachansu injection (HCS), Fufang Kushen injection (FFKS), Tongguanteng injection (TGT), Yadanzi injection (YDZ), Shenqi Fuzheng injection (SQFZ), Polyporus umbellatus polysaccharide injection (PUP), Kangai injection (KA), Kanglaite injection (KLT), and Astragalus polysaccharide (APS). Patients who received systemic or intravenous chemotherapy other than intrapleural DDP, or oral TCM formulas, or other TCM interventions in addition to the above 10 CHIs were excluded.

#### 2.2.4 Types of outcomes

We used the following dichotomous outcomes for easier interpretation into clinical guidance. The primary outcome was the clinical effective rate for MPE, defined as the proportion of patients achieving complete response (CR) and partial response (PR) after treatment according to the World Health Organization criteria (16, 17), which could be computed as the number of patients achieving CR and PR divided by the total number of patients treated. Secondary outcomes were the rate of Karnofsky performance status (KPS) improvement (referring to KPS score increasing more than 10 points after treatment) and incidence of adverse events including gastrointestinal reactions, leukopenia, chest pain, and fever.

### 2.3 Study selection and data extraction

EndNote (EN) X9.3.3 was used to manage literature. One review author (YFX) excluded ineligible studies first by screening titles and abstracts. This was followed by two review authors (YRC and YFX) independently identifying eligible studies through full-text review. Disagreements were resolved through discussion or by referral to a third author (ZLL).

Two review authors (YFX and YXS) independently extracted data from eligible studies. Data were cross-checked for accuracy, and disagreements were resolved through discussion. The following data items were extracted: (1) publication information including first author and year of publication; (2) study characteristics including sample size, follow-up duration, randomization procedure, and blinding procedure; (3) patient characteristics including age and sex; (4) intervention and comparator characteristics including dose and course; and (5) outcome measurements.

### 2.4 Risk of bias assessment

Two authors (YXS and CYL) independently assessed risk of bias for each study using the revised Cochrane risk-of-bias tool for randomized trials (RoB 2) (18). The following five domains were assessed within each included study under the official guidance document (19): (1) bias arising from the randomization process, (2) bias due to deviations from intended interventions, (3) bias due to missing outcome data, (4) bias in measurement of the outcome, and (5) bias in selection of the reported result. An overall risk-of-bias judgment was made on each study as “low risk of bias”, “some concerns”, or “high risk of bias”. Disagreements were resolved through discussion or by consulting a third author (DMQ) for consensus.

### 2.5 Quality of evidence assessment

Two review authors (BFL and YBH) independently assessed the confidence in the body of evidence using the Confidence in Network Meta-Analysis (CINeMA) web application, recommended by the Cochrane handbook for undertaking NMA (20). Disagreements were discussed mutually or by inviting a third author (JPL) to reach a consensus. The methodological framework of CINeMA evaluates confidence in the NMA findings based on the contribution matrix of included studies with consideration of the following six domains: within-study bias, reporting bias, indirectness, imprecision, heterogeneity, and incoherence (21).

### 2.6 Statistical analysis

We performed a standard pairwise meta-analysis using STATA 16.0. A Bayesian NMA was conducted using R software 4.1.0 *via* Just Another Gibbs Sampler (JAGS). The BUGSnet package was used in R (22). We calculated the risk ratio (RR) with 95% confidence intervals (CIs) for the rate of clinical effectiveness, KPS improvement, and AEs. A random-effects model was analyzed to estimate effects among multiple comparisons using the Markov chain Monte Carlo (MCMC) method. We set an uninformative prior distribution for four Markov chains running 250,000 iterations (burn-in iterations = 50,000, thinning factor = 1). Convergence was assessed by the Brooks-Gelman-Rubin diagnosis plot and potential scale reduction factor (PSRF), with a PSRF value close to 1 indicating convergence (23). For the dichotomous outcome measurements among mixed comparisons, RR with 95% credible intervals (CrIs) were presented within league tables. We also calculated surface under the cumulative ranking curve (SUCRA) probability values to estimate rankings of competing interventions. The BUGSnet R package was used to draw SUCRA plots. In our study, higher SUCRA values reflect a higher associated clinical effective rate, higher KPS improvement rate, and a lower rate of adverse events. A network geometry plot was drawn to summarize the treatment network using STATA. Each node represents an intervention, and each edge represents a head-to-head comparison between two different interventions (24). The sizes of nodes and edges display the numbers of patients receiving the treatment and the number of studies for the comparison, respectively (24). We split three-arm studies into two pairwise comparisons by equally dividing the number of patients receiving DDP. Since there were no “closed loops” in the network plot, we were unable to assess inconsistency among direct and indirect comparisons. Statistical heterogeneities were tested using the χ2 test with a significance level of 0.1 and quantified using *I* ^2^ statistics. Substantial heterogeneities were considered with *I* ^2^ greater than 50%. There was insufficient information in included studies for conducting subgroup analysis considering different lung cancer subtypes or treatment duration. A subgroup analysis considering different doses of DDP was conducted to identify substantial sources of clinical heterogeneity. Comparison-adjusted funnel plots were presented to assess small study effects and potential publication bias using STATA.

## 3 Results

### 3.1 Search results

**Figure 1:**
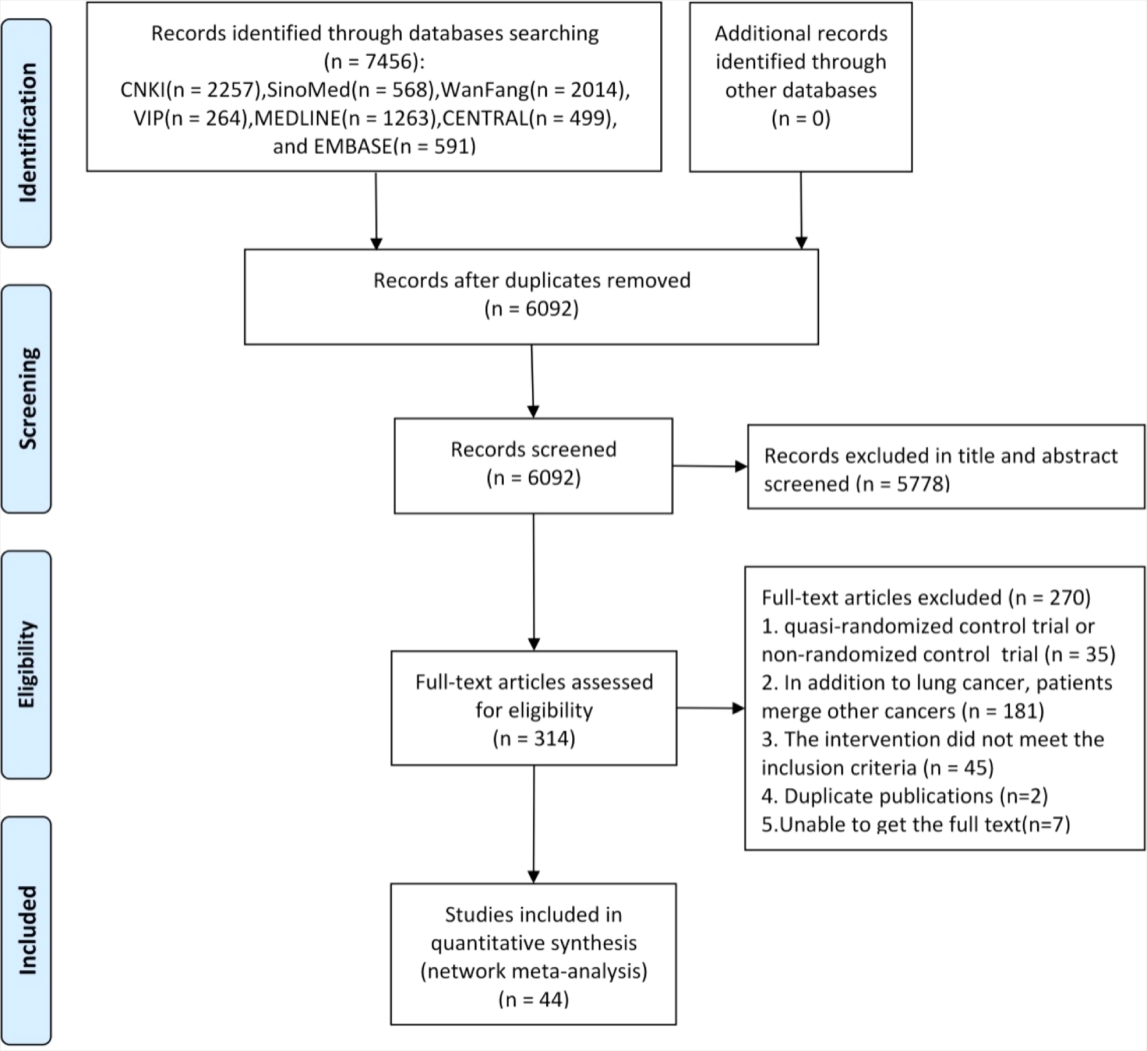
Flowchart of the search for eligible studies. Note: n, number of articles. CNKI, China National Knowledge Infrastructure; SinoMed, the Chinese Biomedical Literature Database; WanFang, the WanFang Database; VIP, the Chinese Scientific Journals Full-Text Database. n, number of articles. CNKI, China National Knowledge Infrastructure; SinoMed, the Chinese Biomedical Literature Database; WanFang, the WanFang Database; VIP, the Chinese Scientific Journals Full-Text Database.

A total of 7,456 citations were identified from seven databases. After removing 1,364 duplicates, a further 5,778 were excluded due to irrelevancy based on their titles and abstracts. The full text of the remaining 314 studies was screened, of which 44 RCTs were deemed eligible. The PRISMA flow diagram for the study selection process is shown in **Figure 1**.

### 3.2 Characteristics of included studies

A total of 2,573 lung cancer patients and 6 kinds of CHIs were involved in the 44 RCTs in which all the patients were in advanced stage. The average age of patients in the vast majority of included studies fluctuated between 50 and 70. All patients received treatment for at least 2 weeks. In terms of treatment, 1,258 patients used DDP alone, 1,096 patients were treated with CHIs combined with DDP, and 219 patients received only CHIs. For the outcomes, 43 studies (97.7%) reported clinical effective rate, 30 studies (68.2%) evaluated the improvement rate of KPS score, and 33 studies (75.0%), 25 studies (56.8%), 26 studies (59.1%), 21 studies (47.7%) assessed the incidence of gastrointestinal reactions, leukopenia, chest pain, and fever, respectively. Details of the baseline characteristics of the studies are shown in **Table 1**.

**Table 1:** Characteristics of the included studies.

Study ID	Sample size (E/C)		Mean/median age (E/C)	Sex (M/F) (E/C)			Treatment of experiment group^†^	Treatment of control group^†^	Course	Outcomes
Zhu Y, 2011 (25)	43/30		58.4/58.2	(30/13)/(20/10)			DDP 30 mg/m^2^ + AD 70 ml	DDP 30 mg/m^2^	Once a week/×3	①②③⑤⑥
Wang XH, 2010 (26)	30/30		/	(22/8)/(25/5)			DDP 40 mg + AD 50 ml	DDP 40 mg	Once a week/×4	①④
Sun SL, 2012 (27)	21/19		62/60	(14/7)/(13/6)			DDP 20–30 mg/m^2^ + AD 50–80 ml	DDP 20–30 mg/m^2^	Once a week/×4	①②③④⑤⑥
Meng ZL, 2009 (28)	22/20		68	(14/8)/(14/6)			DDP 20–30 mg/m^2^ + AD 50–80 ml	DDP 20–30 mg/m^2^	Once a week/×4	①②③④⑤⑥
Wang Y, 2017 (29)	32/32		(56.7 ± 4.3)/(56.1 ± 4.4)	(22/10)/(23/9)			DDP 20–30 mg/m^2^ + AD 50–80 ml	DDP 20–30 mg/m^2^	Once a week/×4	①②③④⑤⑥
Zhang ZL, 2010 (30)	38/36		46–75	43/31			DDP 40–60 mg + AD 50–70 ml	DDP 40–60 mg	Twice a week/×4	①③⑤⑥
Han ZQ, 2012 (31)	28/28		(62 ± 3)/(58 ± 12)	35/21			DDP 20–40 mg + FFKS 30–50 ml	DDP 20–40 mg	Once a week/×(2–4)	①②③④⑤
Tang XQ, 2018 (32)	30/30		(55.6 ± 2.1)/(53.2 ± 1.8)	/			DDP 40 mg + FFKS 60 ml	DDP 40 mg	Twice a week/×6	①②③④
He L, 2010 (33)	24/20		58/60	(16/8)/(9/11)			DDP 40 mg/m^2^ + FFKS 40 ml	DDP 40 mg/m^2^	Once a week/×3	①③④
Li YP, 2009 (34)	30/30		55/56	(25/5)/(24/6)			DDP 40 mg + FFKS 20 ml	DDP 40 mg	Once a week	①③④⑤⑥
Wu CY, 2019 (35)	25/25		(53.48 ± 4.26)/(55.14 ± 5.32)	(16/9)/(14/11)			DDP 40–60 mg + FFKS 40–60 ml	DDP 40–60 mg	Once or twice every 2 weeks	①②③
Liu L, 2017 (36)	30/30		56.4/54.2	/			DDP 40 mg + FFKS 20 ml	DDP 40 mg	Once a week/×4	①②③④
Shi WJ, 2017 (37)	30/30		(56.8 ± 5.7)/(56.4 ± 5.8)	(18/12)/(21/9)			DDP 40 mg + FFKS 20 ml	DDP 40 mg	Once a week/×4	①②③④
Liu SY, 2017 (38)	32/32		(56 ± 1)/(55 ± 1)	(18/14)/(17/15)			DDP 60 mg + HCS 20 ml	DDP 60 mg	Once a week/×2	①③④⑤⑥
Qu DM, 2012 (39)	24/22		63	(15/9)/(12/10)			DDP 40–60 mg + KA 50 ml	DDP 40–60 mg	Once a week/×3	①②
He JY, 2011 (40)	20/20		58.2	24/16			DDP 80 mg + KA 60 ml	DDP 80 mg	Once a week/×6	①③④
Li HH, 2012 (41)	30/30		35–78	38/22			DDP 50 mg + KLT 100 ml	DDP 50 mg	Once a week/×2	①②
Pan JJ, 2007 (42)	36/34		60 ± 21	(22/14)/(21/13)			DDP 40 mg + FFKS 30 ml	DDP 40 mg	Once a week/×3	①②④
Yang DF, 2015 (43)	25/25		(62.2 ± 2.6)/(61.2 ± 2.3)	(16/9)/(17/8)			DDP 25 mg + AD 75 ml	DDP 25 mg	Once a week/×5	③④
Shen SL, 2017 (44)	40/40		(64.6 ± 4.7)/(62.5 ± 5.2)	(23/17)/(29/11)			DDP 50 mg/m^2^ + YDZ 50 ml	DDP 50 mg/m^2^	Once a week/×4	①②③④⑤⑥
Liu D, 2015 (45)	46/42		60.2 ± 8.2	48/40			DDP 40–60 mg + FFKS 20 ml	DDP 40–60 mg	Once a week/×3	①②③⑤⑥
Wu MB, 2020 (46)	18/18		(67.37 ± 3.5)/(65.33 ± 4.1)	(11/7)/(14/4)			DDP 25 mg/m^2^ + AD 50 ml	DDP 25 mg/m^2^	Once a week/×(12–18)	①③④⑤⑥
Jing Y, 2017 (47)	30/33		63.84 ± 1.59	(18/12)/(16/17)			DDP 40 mg/m^2^ + YDZ 60 ml	DDP 40 mg/m^2^	Once a week/×2	①②
Peng HY, 2020 (48)	25/25		(57.2 ± 2.1)/(56.9 ± 1.9)	(14/11)/(15/10)			DDP 30 mg/m^2^ + FFKS 40 ml	DDP 30 mg/m^2^	Three times every 2 weeks/×2	①③⑤⑥
Mo SX, 2009 (49)	28/28		50.3/51.8	(17/11)/(18/10)			DDP 80–100 mg + YDZ 60–80 ml	DDP 80–100 mg	Once a week/×3	①②③④⑤⑥
Liu Y, 2014 (50)	14/14		45–85	18/10			DDP 40 mg + YDZ 40 ml	DDP 40 mg	Five times a week/×4	①②
Song YJ, 2011 (51)	30/30/30		56 ± 11.5	53/37			DDP 40 mg/m^2^ + YDZ 50 ml; YDZ50 ml	DDP 40 mg/m^2^	Once a week/×4	①②
Wang HM, 2007 (52)	35/35		58	45/25			DDP 20–30 mg/m^2^ + YDZ 80–100 ml	DDP 20–30 mg/m^2^	Once every 5–7 days/×4	①②③④⑤⑥
Zhang SF, 2009 (53)	27/23	72			(19/8)/(16/7)	DDP 20–30 mg/m^2^ + YDZ 50–100 ml		DDP 20–30 mg/m^2^	Once every 5–7 days/×4	①②③④⑤⑥
Liu B, 2012 (54)	32/32	57.2			31/33	DDP 40 mg/m^2^ + YDZ 100 ml		DDP 40 mg/m^2^	Once every 5–7 days/×4	①②③④⑤⑥
Guo YF, 2013 (55)	34/28	63/68			(24/10)/(22/6)	DDP 60–80 mg + YDZ 60 ml		DDP 60–80 mg	Once a week/×(2-3)	①②
Zhang H, 2013 (56)	34/30	62.5/56			(28/6)/(24/6)	DDP 40–60 mg + YDZ 40–50 ml		DDP 40–60 mg	Once a week/×3	①⑤
Wang CY, 2016 (57)	30/30	(60.25 ± 1.64)/(63.84 ± 1.59)			(18/12)/(16/14)	DDP 40 mg/m^2^ + YDZ 60 ml		DDP 40 mg/m^2^	Once a week/×2	①②
Chen SL, 2015 (58)	30/30	(56.6 ± 11.9)/(57.7 ± 12.5)			(18/12)/(17/13)	DDP 20–30 mg/m^2^ + YDZ 60–90 ml		DDP 20–30 mg/m^2^	Once a week/×3	①②③④⑤⑥
Huang XM, 2007 (59)	20/18	55/56			(15/5)/(13/5)	DDP 40 mg + FFKS 20 ml		DDP 40 mg	Once a week	①③④⑤⑥
Wang XC, 2014 (60)	32/32	68			46/18	DDP 60 mg + AD 40 ml		DDP 60 mg	Once a week/×4	①③④⑤
Liu CX, 2013 (61)	56/56	(62.18 ± 8.95)/(62.05 ± 9.05)			(28/28)/(29/27)	DDP 30 mg + AD 100 ml		DDP 30 mg	Once a week/×4	①②③④⑤
Zhang HZ, 2015 (62)	32/26	45–72/47–76			(23/9)/(18/8)	KLT 200 ml		DDP 30 mg	Five times a week/×4	①③⑤⑥
Sun LH, 2005 (63)	25/25	32–74			23/27	AD 50 ml		DDP 40 mg	Twice a week/×4	①②③⑤⑥
Hu Q, 2008 (64)	20/20	(64.5 ± 2.3)/(64.3 ± 2.1)			(13/7)/(12/8)	FFKS 20 ml		DDP 30 mg	Once a day/×3	①②③④⑤⑥
Fu J, 2005 (65)	20/20	35–74			/	AD 50 ml		DDP 40 mg	Twice a week/×4	①
Xing HM, 2013 (66)	45/42	(60.2 ± 7.9)/(62.5 ± 8.4)			(28/17)/(24/18)	FFKS 20 ml		DDP 40–60 mg	Once every 3–5 days/×4	①②③⑤⑥
Wang K, 2010 (67)	21/21	32–75			/	YDZ 50 ml		DDP 40 mg	Once 2 weeks/×2	①②③
Wang JH, 2013 (68)	26/26	58.85/58.88			(15/11)/(14/12)	AD 100 ml		DDP 80–100 mg	Once a week/×(2–4)	①②③⑤

^†^All treatments were administered through intrapleural injection. E, experiment group; C, control group; M, male; F, female; DDP, cisplatin; AD, Aidi injection; FFKS, Fufangkushen injection; HCS, Huachansu injection; KA, Kangai injection; KLT, Kanglaite injection; YDZ, Yadanzi injection. ① Clinical effective rate; ② The improvement rate of KPS score; ③ Incidence of gastrointestinal reactions; ④ Incidence of Leukopenia; ⑤ Incidence of chest pain; ⑥ Incidence of fever.

Of the 44 RCTs included, all were two-arm studies except for one (51) three-arm study. The three-arm study administered YDZ combined with DDP, DDP alone, and YDZ alone. The interventions for all the two-arm studies were either combined therapies of CHIs and DDP or CHIs alone, compared to DDP alone. Among the combined therapies, there were six kinds of CHIs: AD combined with DDP [10 RCTs (25–30, 43, 46, 60, 61)], FFKS combined with DDP [11 RCTs (31–37, 42, 45, 48, 59)], HCS combined with DDP [one RCT (38)], KA combined with DDP [two RCTs (39, 40)], KLT combined with DDP [one RCT (41)], and YDZ combined with DDP [12 RCTs (44, 47, 49–58)]. As for the studies that used CHIs alone, there were four kinds of CHIs: KLT [one RCT (62)], AD [three RCTs (63, 65, 68)], FFKS [two RCTs (64, 66)], and YDZ [two RCTs (51, 67)]. The detailed information about compositions, indications, and mechanisms of the CHIs is described in **Supplementary File S3**.

### 3.3 Risk of bias assessment

Considering the bias generated by the randomization process, all studies had adopted a randomized approach, and reported that the baselines of the two groups were comparable. However, due to the lack of specified methods for generating allocation sequence and concealment, 41 of 44 RCTs were assessed as “some concerns”. Two RCTs (45, 56) were classified as low risk with envelopes for concealment and double-blind procedure mentioned, respectively. One RCT (60) was classified as high risk because of collecting data retrospectively. About the bias due to deviations from intended interventions, all included studies reported no deviations from allocated interventions and used an appropriate method to analyze treatment effects. Thus, all studies were regarded as “low risk”. In terms of bias due to missing outcome data and bias in measurement of the outcome, we could get complete data in all studies; moreover, the measurement or determination of the outcomes in the two groups is consistent and objective; hence, all studies were evaluated as “low risk”. As for the bias in selection of the reported results, there were no pre-reported study protocols identified; thus, all RCTs were rated as “some concerns”. Details of the risk of bias assessment are shown in **Supplementary File S4**.

### 3.4 Pairwise meta-analysis

We performed a direct comparison of interventions with different CHIs compared with DDP in the six outcomes. The forest plot and detailed information of the heterogeneity analysis for the six outcomes are shown in **Supplementary File S5**. Most of the comparisons between the two groups showed no significant heterogeneity, except for FFKS compared to DDP for clinical effective rate (*I* ^2^ = 69%), YDZ compared to DDP for the improvement rate of KPS score (*I* ^2^ = 90.9%), and FFKS+DDP compared to DDP for the incidence of gastrointestinal reactions (*I* ^2^ = 59.5%). Thus, the fixed-effects model for meta-analysis was used. Subgroup analysis and sensitivity analysis was conducted when there was heterogeneity. Since the tumor stages included in this study were all stage IV, which were consistent and had no obvious clinical heterogeneity, and different doses and courses of chemotherapy may be substantial sources of clinical heterogeneity, a subgroup analysis conducted on the total dose of DDP with sufficient studies indicated that the dose was the likely cause of the heterogeneity. Changing the effect model and eliminating the literature effect size one by one revealed that the original results were not changed (*p* < 0.05), indicating that the sensitivity analysis results were negative, and the results were relatively robust and reliable. The details of subgroup analysis and sensitivity analysis are shown in **Supplementary File S6**.

### 3.5 Network meta-analysis

**Figure 2:**
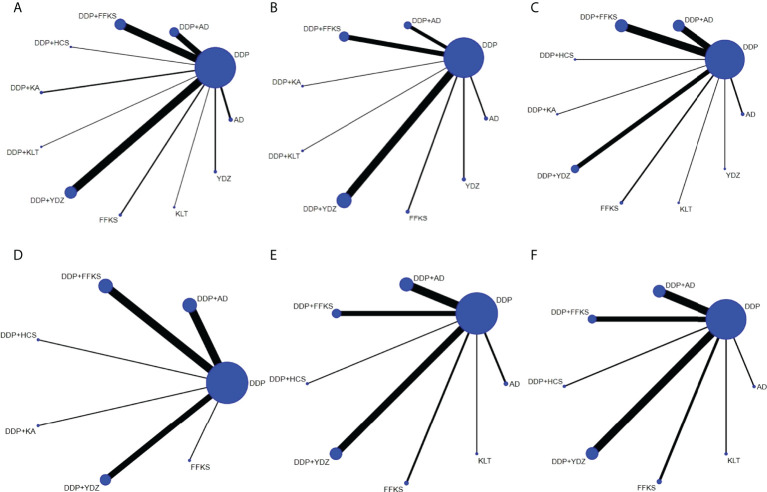
The network graphs comparing CHIs for lung cancer with MPE. **(A)** Clinical effective rate. **(B)** The improvement rate of KPS score. **(C)** Incidence of gastrointestinal reactions. **(D)** Incidence of leukopenia. **(E)** Incidence of chest pain. **(F)** Incidence of fever. Each node represents an intervention, and each edge represents a head-to-head comparison between two different interventions. The sizes of nodes and edges display the numbers of patients receiving the treatment and the number of studies for the comparison, respectively. AD, Aidi injection; DDP, cisplatin; FFKS, Fufang Kushen injection; HCS, Huachansu injection; KA, Kangai injection; KLT, Kanglaite injection; YDZ, Yadanzi injection.

Network graphs comparing CHIs for lung cancer patients with MPE in each of the six outcomes are shown in **Figure 2**. The network graphs were generated using Stata 16.0. Each intervention was shown by a circular node, and each connection represented a contrast. The diameter of the circular node was positively correlated with the number of patients included, and line thickness was positively related to the number of direct comparisons.

It can be seen from **Figure 2** that DDP was used as the comparator arm in all studies, but as there was no direct comparison between any two interventions, no closed loop existed. As a result, an inconsistency test was not required for this study. Based on the heterogeneity results and the baseline data of the studies shown in **Table 2**, we believe that the homogeneity and similarity assumptions between the studies were sufficient in the NMA, and therefore, the consistency model and random-effects model were chosen to build Bayesian models. The maximum number of iterative calculations during the model building process was 250,000.

**Table 2:** League table of NMA estimations.


**Table 2A Network meta-analysis comparisons for clinical effective rate**																							
**DDP**																							
0.91 (0.73,1.13)		**AD**																					
**0.72 (0.63,0.80)**		0.79 (0.62,1.01)		**DDP+AD**																			
**0.73 (0.65,0.81)**		0.80 (0.63,1.03)		1.02 (0.87,1.20)		**DDP+FFKS**																	
**0.60 (0.38,0.87)**		0.65 (0.40,1.02)		0.83 (0.52,1.24)		0.81 (0.51,1.21)		**DDP+HCS**															
**0.71 (0.48,1.00)**		0.78 (0.50,1.18)		0.99 (0.66,1.43)		0.97 (0.65,1.40)		1.19 (0.69,2.09)		**DDP+KA**													
**0.63 (0.41,0.89)**		0.69 (0.43,1.05)		0.89 (0.57,1.27)		0.87 (0.56,1.24)		1.06 (0.61,1.87)		0.89 (0.52,1.50)			**DDP+KLT**										
**0.69 (0.62,0.77)**		**0.76 (0.60,0.97)**		0.96 (0.82,1.13)		0.94 (0.81,1.10)		1.16 (0.78,1.84)		0.97 (0.68,1.45)			1.09 (0.76,1.69)			**DDP+YDZ**							
0.82 (0.64,1.04)		0.90 (0.65,1.25)		1.15 (0.87,1.50)		1.12 (0.85,1.47)		1.38 (0.87,2.29)		1.16 (0.75,1.83)			1.30 (0.84,2.11)			1.19 (0.90,1.55)			**FFKS**				
0.78 (0.51,1.14)		0.86 (0.53,1.33)		1.09 (0.70,1.61)		1.07 (0.69,1.58)		1.32 (0.74,2.31)		1.10 (0.64,1.88)			1.24 (0.71,2.14)			1.13 (0.73,1.67)			0.95 (0.58,1.49)	**KLT**			
0.92 (0.66,1.26)		1.01 (0.69,1.48)		1.29 (0.91,1.80)		1.26 (0.89,1.76)		1.55 (0.93,2.63)		1.30 (0.80,2.12)			1.46 (0.90,2.47)			1.34 (0.95,1.86)			1.13 (0.74,1.67)	1.18 (0.72,1.99)		**YDZ**	
**Table 2B Network meta-analysis comparisons for the improvement rate of KPS score**																							
**DDP**																							
**0.63 (0.43,0.89)**		**AD**																					
**0.68 (0.56,0.81)**		1.07 (0.73,1.65)		**DDP+AD**																			
**0.67 (0.56,0.79)**		1.06 (0.72,1.62)		0.99 (0.76,1.26)		**DDP+FFKS**																	
0.51 (0.21,1.02)		0.81 (0.32,1.79)		0.76 (0.30,1.54)		0.77 (0.31,1.55)		**DDP+KA**															
0.73 (0.50,1.04)		1.17 (0.69,1.97)		1.08 (0.71,1.62)		1.10 (0.72,1.63)		1.43 (0.65,3.71)		**DDP+KLT**													
**0.68 (0.60,0.76)**		1.08 (0.75,1.63)		1.00 (0.80,1.25)		1.01 (0.83,1.25)		1.33 (0.66,3.27)		0.92 (0.64,1.38)			**DDP+YDZ**										
0.79 (0.54,1.12)		1.26 (0.76,2.12)		1.17 (0.77,1.73)		1.19 (0.79,1.77)		1.56 (0.71,3.98)		1.08 (0.65,1.81)			1.17 (0.79,1.69)			**FFKS**							
0.76 (0.44,1.30)		1.21 (0.63,2.37)		1.12 (0.63,1.99)		1.14 (0.64,2.01)		1.50 (0.62,4.08)		1.04 (0.54,2.02)			1.12 (0.64,1.94)			0.96 (0.51,1.85)			**YDZ**				
**Table 2C Network meta-analysis comparisons for incidence of gastrointestinal reactions**																							
**DDP**																							
**12.79 (2.92,105.93)**		**AD**																					
**2.46 (1.46,4.28)**		**0.19 (0.02,0.94)**		**DDP+AD**																			
**2.40 (1.55,3.96)**		**0.19 (0.02,0.90)**		0.97 (0.48,2.01)		**DDP+FFKS**																	
0.97 (0.19,4.89)		**0.07 (0.01,0.68)**		0.39 (0.07,2.14)		0.40 (0.07,2.13)		**DDP+HCS**															
3.25 (0.55,29.52)		0.25 (0.02,3.56)		1.32 (0.20,12.66)		1.35 (0.21,12.67)		3.41 (0.30,50.31)		**DDP+KA**													
1.65 (0.88,3.24)		**0.13 (0.01,0.66)**		0.67 (0.29,1.57)		0.69 (0.31,1.53)		1.71 (0.30,9.91)		0.51 (0.05,3.45)			**DDP+YDZ**										
**16.81 (3.83,130.04)**		1.32 (0.10,16.24)		**6.85 (1.40,56.08)**		**7.00 (1.46,56.24)**		**17.89 (1.92,230.72)**		5.27 (0.37,77.49)			**10.24 (1.99,85.75)**		**FFKS**								
**6.92 (1.26,61.22)**		0.54 (0.04,7.39)		2.82 (0.46,26.16)		2.89 (0.48,26.26)		7.29 (0.67,105.90)		2.14 (0.13,35.16)			4.21 (0.66,40.02)		0.41 (0.03,5.78)			**KLT**					
**4.90 (1.26,22.92)**		0.38 (0.03,3.23)		2.00 (0.45,10.10)		2.05 (0.47,9.99)		5.12 (0.61,46.95)		1.51 (0.11,15.89)			2.98 (0.65,15.57)		0.29 (0.03,2.47)			0.71 (0.05,7.06)			**YDZ**		
**Table 2D Network meta-analysis comparisons for incidence of leukopenia**																							
**DDP**																							
**1.69 (1.31,2.35)**	**DDP+AD**																						
**2.02 (1.51,2.80)**	1.20 (0.78,1.80)		**DDP+FFKS**																				
**3.87 (1.23,18.55)**	2.28 (0.69,11.03)		1.91 (0.58,9.37)		**DDP+HCS**																		
**2.98 (1.15,10.21)**	1.76 (0.64,6.17)		1.48 (0.54,5.20)		0.78 (0.13,4.13)		**DDP+KA**																
**2.30 (1.58,3.47)**	1.36 (0.82,2.20)		1.14 (0.69,1.88)		0.60 (0.12,2.03)		0.77 (0.21,2.18)		**DDP+YDZ**														
**10.24 (1.74,236.98)**	**6.07 (1.00,138.93)**		5.06 (0.84,116.98)		2.66 (0.25,71.19)		3.40 (0.39,88.58)		4.48 (0.72,104.55)			**FFKS**											
**Table 2E Network meta-analysis comparisons for incidence of chest pain**																							
**DDP**																							
**2.48 (1.09,6.42)**	**AD**																						
**1.88 (1.08,3.09)**	0.76 (0.25,1.96)		**DDP+AD**																				
**1.99 (1.01,4.16)**	0.80 (0.25,2.43)		1.06 (0.46,2.69)		**DDP+FFKS**																		
2.41 (0.92,7.07)	0.97 (0.25,3.75)		1.28 (0.45,4.40)		1.21 (0.36,4.27)		**DDP+HCS**																
1.15 (0.70,2.06)	0.46 (0.16,1.27)		0.61 (0.30,1.41)		0.58 (0.24,1.42)		0.48 (0.15,1.50)		**DDP+YDZ**														
**9.44 (3.39,35.14)**	3.83 (0.94,17.82)		**5.04 (1.64,20.84)**		**4.75 (1.34,20.55)**		3.93 (0.88,19.99)		**8.24 (2.48,32.83)**		**FFKS**												
2.45 (0.79,8.66)	0.99 (0.22,4.49)		1.30 (0.38,5.26)		1.23 (0.32,5.16)		1.01 (0.21,4.94)		2.13 (0.58,8.05)		0.26 (0.05,1.33)			**KLT**									
**Table 2F Network meta-analysis comparisons for incidence of fever**																							
**DDP**																							
3.78 (0.26,143.83)	**AD**																						
1.19 (0.48,4.99)	0.32 (0.01,7.11)		**DDP+AD**																				
**3.24 (1.04,17.45)**	0.87 (0.02,21.53)		2.72 (0.51,15.21)		**DDP+FFKS**																		
1.65 (0.27,10.50)	0.43 (0.01,11.05)		1.41 (0.12,9.14)		0.52 (0.04,3.83)		**DDP+HCS**																
1.15 (0.48,3.54)	0.31 (0.01,5.71)		0.97 (0.19,3.63)		0.36 (0.06,1.58)		0.69 (0.10,6.17)		**DDP+YDZ**														
2.86 (0.76,11.87)	0.76 (0.02,15.88)		2.42 (0.31,11.64)		0.89 (0.10,4.93)		1.74 (0.18,17.00)		2.50 (0.42,12.45)		**FFKS**												
1.82 (0.26,13.67)	0.47 (0.01,13.65)		1.54 (0.12,11.98)		0.56 (0.04,5.01)		1.10 (0.08,16.15)		1.58 (0.16,13.18)		0.63 (0.06,7.02)			**KLT**									

The differences between the compared groups were deemed as significant when the 95% CrI of the RR did not contain 1.00, which is marked as bold font. The data are the RR (95% CrI) of the column intervention compared to the row intervention, i.e., for the clinical effective rate, DDP alone was significantly less effective than DDP plus AD (RR 0.72, 95% CrI 0.63–0.80). DDP, cisplatin; AD, Aidi injection; FFKS, Fufangkushen injection; HCS, Huachansu injection; KA, Kangai injection; KLT, Kanglaite injection; YDZ, Yadanzi injection.

**Figure 3:**
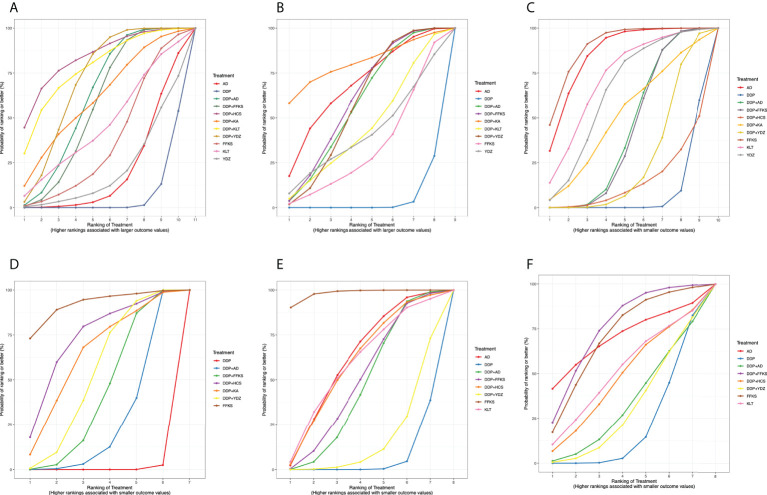
Surface under the cumulative ranking curve (SUCRA) probabilities of different interventions for six outcomes. **(A)** Clinical effective rate. **(B)** The improvement rate of KPS score. **(C)** Incidence of gastrointestinal reactions. **(D)** Incidence of leukopenia. **(E)** Incidence of chest pain. **(F)** Incidence of fever. The area under each curve corresponds to the probability of each treatment being the best treatment. AD, Aidi injection; DDP, cisplatin; FFKS, Fufang Kushen injection; HCS, Huachansu injection; KA, Kangai injection; KLT, Kanglaite injection; YDZ, Yadanzi injection.

RRs (95% CrIs) of all interventions for the six outcomes in our NMA are shown in **Table 2**. The results of the ranking probabilities based on SUCRA are shown in **Table 3** and **Figure 3**. We also provided the rankograms in **Figure S7**.

**Table 3:** Ranking probability of interventions.

Intervention	Clinical effective rate		The improvement rate of KPS score		Incidence of gastrointestinal reactions		Incidence of leukopenia		Incidence of chest pain		Incidence of fever	
	SUCRA (%)	Rank	SUCRA (%)	Rank	SUCRA (%)	Rank	SUCRA (%)	Rank	SUCRA (%)	Rank	SUCRA (%)	Rank
DDP+AD	62.80	4	58.02	4	43.88	6	25.98	6	46.68	6	33.19	6
DDP+FFKS	57.34	6	60.87	3	42.79	7	42.34	5	50.36	5	75.45	1
DDP+HCS	84.33	1	–	–	14.56	9	72.64	2	59.97	3	47.95	5
DDP+KA	61.25	5	80.82	1	51.43	5	63.62	3	–	–	–	–
DDP+KLT	78.15	2	44.95	6	–	–	–	–	–	**-**	–	**-**
DDP+YDZ	71.45	3	57.93	5	26.80	8	53.17	4	17.08	7	31.00	7
KLT	46.90	7	–	–	72.26	3	–	–	59.45	4	51.21	4
AD	21.24	10	68.37	2	85.67	2	–	–	62.05	2	69.87	3
YDZ	21.41	9	41.52	7	64.91	4	–	–	–	–	–	–
FFKS	38.09	8	33.46	8	89.92	1	91.85	1	98.17	1	70.67	2
DDP	7.06	11	4.03	9	7.77	10	0.42	7	6.21	8	20.67	8

DDP, cisplatin; AD, Aidi injection; FFKS, Fufangkushen injection; HCS, Huachansu injection; KA, Kangai injection; KLT, Kanglaite injection; YDZ, Yadanzi injection.

#### 3.5.1 Clinical effective rate

A total of 43 studies reported the clinical effective rate, including the three-arm study. There were 11 interventions involved in this NMA where DDP was used as a common control to indirectly compare the clinical effectiveness of different CHIs.

**Table 2A** details the effectiveness of the comparison of different interventions by RRs and the corresponding 95% CrIs in NMA. The combination therapy of CHIs and DDP was significantly more effective in improving the clinical effective rate than DDP alone. However, CHIs alone did not show statistical significance compared with DDP alone. The results of the SUCRA showed that the combination of HCS and DDP might be associated with the highest probability of being the best choice for improving the clinical effective rate (84.33%) and DDP alone showed the lowest probability (7.06%). The probability ranked in the middle was the CHIs alone.

#### 3.5.2 The improvement rate of KPS score

There were 30 studies that informed the improvement rate of KPS score, including the three-arm study, and nine related interventions. The network comparisons displayed in **Table 2B** suggested that there were four interventions (AD, DDP+AD, DDP+FFKS, and DDP+YDZ) that could improve KPS compared to DDP alone, though other interventions showed no statistical significance.

According to the SUCRA probabilities, the ranking of interventions to improve the KPS score is as follows: DDP+KA (80.82%) > AD (68.37%) > DDP+FFKS (60.87%) > DDP+AD (58.02%) > DDP+YDZ (57.93%) > DDP+KLT (44.95%) > YDZ (41.52%) > FFKS (33.46%) > DDP (4.03%). As with clinical effective rate, DDP alone might show the lowest probability of improving KPS scores.

#### 3.5.3 Incidence of gastrointestinal reactions

In terms of the incidence of adverse events, 33 studies involving 10 interventions reported incidence of gastrointestinal reactions. Network comparisons suggested that six types of treatment (DDP+ AD, DDP+FFKS, AD, FFKS, KLT, and YDZ) were better than DDP alone in reducing the incidence of gastrointestinal reactions.

As the results of SUCRA show, four CHIs (FFKS, AD, KLT, and YDZ) when used alone might have minimal incidence of gastrointestinal reactions, and CHIs combined with DDP could reduce the incidence of gastrointestinal reactions compared to DDP alone.

#### 3.5.4 Incidence of leukopenia

A total of 25 studies involving seven interventions showed incidence of leukopenia. Regardless of whether CHIs were combined or used by itself, the use of CHIs showed a lower incidence of leukopenia than DDP alone.

Similar to the incidence of gastrointestinal reactions, the lowest incidence of leukopenia was seen when using FFKS, and CHIs combined with DDP could reduce adverse events. The rank probability was as follows: FFKS (91.85%), DDP+HCS (72.64%), DDP+KA (63.62%), DDP+YDZ (53.17%), DDP+FFKS (42.34%), DDP+AD (25.98%), and DDP (0.42%).

#### 3.5.5 Incidence of chest pain

A total of 26 studies involving eight interventions, reported incidence of chest pain. Four types of treatment (AD, DDP+AD, DDP+FFKS, and FFKS) showed a lower incidence of chest pain than DDP alone, while other treatments did not show statistical significance compared with DDP alone.

According to the rank probabilities, FFKS might have the highest possibility of showing less incidence in chest pain (98.17%), while DDP alone might be the least improved treatment (6.21%).

#### 3.5.6 Incidence of fever

A total of 21 studies involving eight interventions reported incidence of fever. **Table 2E** reveals that DDP combined with FFKS showed a lower incidence of fever than DDP alone (RR = 3.24, 95% CrI: 1.04–17.45), while others did not show statistical significance compared with DDP alone.

With the incidence of fever, DDP+FFKS might have the highest possibility of showing less incidence in fever (75.45%), and the DDP alone still might be the worst performer (20.67%).

### 3.6 Publication bias

Comparison-adjusted funnel plots were used to detect whether there was publication bias in the six outcomes and are provided in **Supplementary File S8**. It can be seen in **Figure S8** that there are different angles between the calibration auxiliary line and the center line, indicating that this study may have potential publication bias and small study effects in the six outcomes.

### 3.7 Confidence in evidence

The grading of the comparisons with CINeMA displayed mainly “low” to “very low” confidence ratings. This was due to the network without closed loops of evidence (without mixed evidence); hence, inconsistency cannot be assessed. Thus, the “Incoherence” levels were all illustrated as “Some concerns”. There were “Major concerns” about “Imprecision,” usually related to the low numbers of trials available for some comparisons in this study. Details are provided in **Supplementary File S9**.

## 4 Discussion

CHIs are commonly used as a complementary treatment in China. However, due to the lack of direct comparison between different types of CHIs, it is often difficult for clinical physicians to choose the optimal therapy for patients with MPE. As a result, this NMA was undertaken to understand the best available evidence on the comparisons of different types of CHIs, to assist physicians in clinical practice.

### 4.1 Summary of evidence

This NMA evaluated six types of CHIs as adjuvant and four types of CHIs as alternative treatments when compared to DDP alone for lung cancer patients with MPE. The CHIs included AD, FFKS, HCS, KA, KLT, and YDZ. The six outcomes assessed included clinical effective rate, the improvement rate of KPS score, and the incidence of gastrointestinal reactions, leukopenia, chest pain, and fever. The overall heterogeneity between the different comparisons of drugs was found to be low in our NMA. With respect to improvements in clinical effective rate, the NMA results concluded that HCS combined with DDP performed the best. Modern pharmacological studies have shown that cininobufosin and its active compounds (such as bufalin and cininobufosin) have significant antitumor activities and can reverse the regulation of multidrug resistance and immune response. Moreover, some clinical data have indicated that cinocobalamin may have effective anticancer activity, with low toxicity and few adverse effects (69). In the aspect of KPS score, KA combined with DDP might be the best choice. KA is an intravenous fluid made from an extraction of three Chinese herbs (ginseng, astragalus, and matrine), which has a variety of pharmacological effects including antitumor, reductions in adverse reactions caused by chemotherapy, and improvements in the body’s immune function (70). In relation to reducing the incidence of adverse reactions, FFKS alone showed the best results in reducing gastrointestinal reactions, leukopenia, and chest pain, and FFKS combined with DDP demonstrated the best safety when it comes to fever. The main components of FFKS are oxymatrine, matrine, and other alkaloids, which could induce cell apoptosis and enhance the effects of DDP in non-small-cell lung cancer (NSCLC) cells (71), and prevent or reduce chemotherapy- and/or radiotherapy-induced toxicity when combined with chemotherapeutic drugs (72). Apart from this, other CHIs are able to exert their antitumor and reduce side effects through various mechanisms. The AD contains multiple active ingredients, including astragaloside (Re, Rb1, and Rg1), ginsenoside, cantharidin, eleutheroside E, and syringin, which significantly inhibit the proliferation of various tumor cells, induced cell apoptosis, and have shown outstanding antitumor properties, immune regulation functions, and decrease in chemotherapy-related ADRs (73). Coixenolide is the main active ingredient of KLT, which exhibits anticancer and immunomodulatory properties. The induction of NF-κB-mediated gene transcription in CD4+ T cells participates in the immunomodulatory activity of KLT (74). Research has shown that YDZ could induce the death of cancer cells through a variety of mechanisms, and exhibited higher activity and a broader antitumor spectrum *in vitro (* 75).

As the rank probability of six outcomes suggested, CHIs combined with DDP or single-use CHIs were superior than the use of DDP alone in improving the effective rate and KPS score and reducing the incidence of adverse reactions. However, several CHIs did not show statistical significance when compared with DDP alone in the pairwise meta-analysis. Moreover, because of the wide confidence intervals in the NMA due to the small sample size of included patients and the low incidence of adverse events, the rank results need to be carefully considered. One previous simulation study found that the rank probability of the treatment was underestimated when being tested in the largest number of studies in a given network and overestimated for the treatment included in the smallest number of studies. The results can only be reliable when each treatment involved in the analysis has direct evidence or has obvious advantages in effectiveness (76). In this NMA, there was only one RCT of HCS combined with DDP, one RCT of KA combined with DDP, and two RCTs of FFKS alone included where analysis lacked direct comparisons between certain interventions. The grading of the comparisons with CINeMA showed primarily “low” to “very low” confidence ratings, and as a result, the conclusions based on this NMA may not be trustworthy. We suggest clinicians should choose different treatment methods according to the specific requirements of their patients.

### 4.2 Strengths and limitations

In comparison with published research, this is the first NMA, to our knowledge, that compares different CHIs as an adjuvant or alternative treatment to DDP in the treatment of lung cancer patients with MPE (77, 78). Our research has ascendency. Firstly, strict eligibility criteria were used, particularly inclusion of only patients with pleural effusion caused by lung cancer, and DDP as a fixed control. This ensured consistency of the disease conditions and interventions included in the RCTs, which could decrease clinical heterogeneity. Only antitumor drugs listed by the NHSA in the catalog of Chinese patent medicines were included, to ensure conformity with actual clinical usage and provide relevancy for future clinical practice. Furthermore, the six outcome indicators, clinical effective rate, improvement rate of KPS score, and the incidence of gastrointestinal reactions, leukopenia, chest pain, and fever, were chosen on the basis of whether they could provide comprehensive information to recommend as realistic treatment recommendations.

Nevertheless, limitations and shortcomings existed in our research. Firstly, the overall risk of bias was assessed as some concerns. Secondly, the sample size of included studies was relatively small, and the number of qualified studies included were not sufficient. We believe that the credibility of the NMA could be improved if the sample size was increased, and more eligible studies and more RCTs of different types of CHIs were included. In addition, more ranking comparison on dosage and treatment duration could also be considered. Thirdly, as indicated by our results, the network diagram does not form a typical closed loop, such that the research inconsistencies and credibility of our conclusions cannot be checked. Fourthly, long-term survival outcomes are critical for clinical decision-making, and most studies included in our MNA were primarily focused on the short-term therapeutic outcomes due to the relatively limited treatment course and follow-up time. Finally, owing to the limited scope of application of CHIs, all included studies were carried out in China and all patients were Chinese, which may introduce some degree of selection bias to the results. Notably, the Food and Drug Administration (FDA) of the United States approved the clinical trial of KLT in 2001, and a phase II study in patients with advanced pancreatic cancer has been completed in 2014 (79). The Russian Federation approved the clinical trial of KLT in 2002, and KLT has been marketed in Russia since 2005 with a positive response (80). However, the clinical application of KLT still seems limited outside of China with little information being reported officially, and there is no international multicenter study concerning the effect of KLT on MPE. The conclusions drawn from the results, therefore, cannot be generalized on a large scale worldwide.

## 5 Conclusions

Our NMA evaluated the effectiveness and safety of CHIs as an adjuvant or alternative therapy for DDP in the treatment of lung cancer patients with MPE. To our knowledge, this is the first comprehensive NMA study of its kind. The results showed that CHIs alone or combined with DDP could improve clinical effectiveness and quality of life and reduce AEs, compared to DDP alone. HSC and KA, combined with DDP, may be the most effective considering clinical effective rate and improvement of KPS score, respectively. FFKS, either used alone or in combination therapy with DDP, may be the best in reducing AEs. However, high-quality RCTs with larger sample sizes are needed to further corroborate the evidence.

## Data availability statement

The original contributions presented in the study are included in the article/**Supplementary Material**. Further inquiries can be directed to the corresponding authors.

## Author Contributions

Y-FX and Y-RC: conceptualization, methodology, formal analysis, and writing the original draft. F-LB and Y-BH: methodology and supervision. Y-XS and C-YL: visualization and review editing; JS: language editing and supervision. J-PL: methodology and supervision. Z-LL and D-MQ: conceptualization, funding, and project administration. All authors contributed to the article and approved the submitted version.

## Funding

This work was supported by the National Administration of Traditional Chinese Medicine: 2019 Project of building evidence based practice capacity for TCM (No. 2019XZZX-ZL002), and the National Natural Science Foundation of China (No. 81904052).

## Conflict of interest

The authors declare that the research was conducted in the absence of any commercial or financial relationships that could be construed as a potential conflict of interest.

## Publisher’s note

All claims expressed in this article are solely those of the authors and do not necessarily represent those of their affiliated organizations, or those of the publisher, the editors and the reviewers. Any product that may be evaluated in this article, or claim that may be made by its manufacturer, is not guaranteed or endorsed by the publisher.
