# Supplementary material for: Chinese herbal injections versus intrapleural cisplatin for lung cancer patients with malignant pleural effusion: A Bayesian network meta-analysis of randomized controlled trials
Source: Front Oncol. 2022 Sep 20;12:942941. doi: 10.3389/fonc.2022.942941 (PMC9531116; doi:10.3389/fonc.2022.942941)
Supplement: Supplementary file 2 [file DataSheet_2.docx]

Supplementary Material

**Supplementary File S1**

**PRISMA NMA Checklist of Items to Include When Reporting A Systematic Review Involving a Network Meta-analysis**

| **Section/Topic** | **Item #** | **Checklist Item** | **Reported on Page #** |
| --- | --- | --- | --- |
| **TITLE** |  |  |  |
| Title | 1 | Identify the report as a systematic review *incorporating a network meta-analysis (or related form of meta-analysis).* | 1 |
|  |  |  |  |
| **ABSTRACT** |  |  |  |
| Structured summary | 2 | Provide a structured summary including, as applicable:  **Background:** main objectives  **Methods:** data sources; study eligibility criteria, participants, and interventions; study appraisal; and *synthesis methods, such as network meta-analysis.*  **Results:** number of studies and participants identified; summary estimates with corresponding confidence/credible intervals; *treatment rankings may also be discussed. Authors may choose to summarize pairwise comparisons against a chosen treatment included in their analyses for brevity.*  **Discussion/Conclusions:** limitations; conclusions and implications of findings.  **Other:** primary source of funding; systematic review registration number with registry name. | 2 |
|  |  |  |  |
| **INTRODUCTION** |  |  |  |
| Rationale | 3 | Describe the rationale for the review in the context of what is already known*, including mention of why a network meta-analysis has been conducted.* | 2-3 |
| Objectives | 4 | Provide an explicit statement of questions being addressed, with reference to participants, interventions, comparisons, outcomes, and study design (PICOS). | 4-5 |
|  |  |  |  |
| **METHODS** |  |  |  |
| Protocol and registration | 5 | Indicate whether a review protocol exists and if and where it can be accessed (e.g., Web address); and, if available, provide registration information, including registration number. | 4 |
| Eligibility criteria | 6 | Specify study characteristics (e.g., PICOS, length of follow-up) and report characteristics (e.g., years considered, language, publication status) used as criteria for eligibility, giving rationale. *Clearly describe eligible treatments included in the treatment network, and note whether any have been clustered or merged into the same node (with justification).* | 4-5 |
| Information sources | 7 | Describe all information sources (e.g., databases with dates of coverage, contact with study authors to identify additional studies) in the search and date last searched. | 4 |
| Search | 8 | Present full electronic search strategy for at least one database, including any limits used, such that it could be repeated. | S2 |
| Study selection | 9 | State the process for selecting studies (i.e., screening, eligibility, included in systematic review, and, if applicable, included in the meta-analysis). | 5 |
| Data collection process | 10 | Describe method of data extraction from reports (e.g., piloted forms, independently, in duplicate) and any processes for obtaining and confirming data from investigators. | 5 |
| Data items | 11 | List and define all variables for which data were sought (e.g., PICOS, funding sources) and any assumptions and simplifications made. | 4-5 |
| **Geometry of the network** | **S1** | Describe methods used to explore the geometry of the treatment network under study and potential biases related to it. This should include how the evidence base has been graphically summarized for presentation, and what characteristics were compiled and used to describe the evidence base to readers. | 6 |
| Risk of bias within individual studies | 12 | Describe methods used for assessing risk of bias of individual studies (including specification of whether this was done at the study or outcome level), and how this information is to be used in any data synthesis. | 6 |
| Summary measures | 13 | State the principal summary measures (e.g., risk ratio, difference in means). *Also describe the use of additional summary measures assessed, such as treatment rankings and surface under the cumulative ranking curve (SUCRA) values, as well as modified approaches used to present summary findings from meta-analyses.* | 6-7 |
| Planned methods of analysis | 14 | Describe the methods of handling data and combining results of studies for each network meta-analysis. This should include, but not be limited to:   - *Handling of multi-arm trials;* - *Selection of variance structure;* - *Selection of prior distributions in Bayesian analyses; and* - *Assessment of model fit.* | 6-7 |
| **Assessment of Inconsistency** | **S2** | Describe the statistical methods used to evaluate the agreement of direct and indirect evidence in the treatment network(s) studied. Describe efforts taken to address its presence when found. | 7 |
| Risk of bias across studies | 15 | Specify any assessment of risk of bias that may affect the cumulative evidence (e.g., publication bias, selective reporting within studies). | 6 |
| Additional analyses | 16 | Describe methods of additional analyses if done, indicating which were pre-specified. This may include, but not be limited to, the following:   - Sensitivity or subgroup analyses; - Meta-regression analyses; - *Alternative formulations of the treatment network; and* - *Use of alternative prior distributions for Bayesian analyses (if applicable).* | 7 |
|  |  |  |  |
| **RESULTS†** |  |  |  |
| Study selection | 17 | Give numbers of studies screened, assessed for eligibility, and included in the review, with reasons for exclusions at each stage, ideally with a flow diagram. | 7 |
| **Presentation of network structure** | **S3** | Provide a network graph of the included studies to enable visualization of the geometry of the treatment network. | 9 |
| **Summary of network geometry** | **S4** | Provide a brief overview of characteristics of the treatment network. This may include commentary on the abundance of trials and randomized patients for the different interventions and pairwise comparisons in the network, gaps of evidence in the treatment network, and potential biases reflected by the network structure. | 9 |
| Study characteristics | 18 | For each study, present characteristics for which data were extracted (e.g., study size, PICOS, follow-up period) and provide the citations. | 7-8 |
| Risk of bias within studies | 19 | Present data on risk of bias of each study and, if available, any outcome level assessment. | 8 |
| Results of individual studies | 20 | For all outcomes considered (benefits or harms), present, for each study: 1) simple summary data for each intervention group, and 2) effect estimates and confidence intervals. *Modified approaches may be needed to deal with information from larger networks.* | 8-9 |
| Synthesis of results | 21 | Present results of each meta-analysis done, including confidence/credible intervals. *In larger networks, authors may focus on comparisons versus a particular comparator (e.g. placebo or standard care), with full findings presented in an appendix. League tables and forest plots may be considered to summarize pairwise comparisons.* If additional summary measures were explored (such as treatment rankings), these should also be presented. | 9-11 |
| **Exploration for inconsistency** | **S5** | Describe results from investigations of inconsistency. This may include such information as measures of model fit to compare consistency and inconsistency models, *P* values from statistical tests, or summary of inconsistency estimates from different parts of the treatment network. |  |
| Risk of bias across studies | 22 | Present results of any assessment of risk of bias across studies for the evidence base being studied. | 11 |
| Results of additional analyses | 23 | Give results of additional analyses, if done (e.g., sensitivity or subgroup analyses, meta-regression analyses*, alternative network geometries studied, alternative choice of prior distributions for Bayesian analyses,* and so forth). | 8-9 |
|  |  |  |  |
| **DISCUSSION** |  |  |  |
| Summary of evidence | 24 | Summarize the main findings, including the strength of evidence for each main outcome; consider their relevance to key groups (e.g., healthcare providers, users, and policy-makers). | 12-13 |
| Limitations | 25 | Discuss limitations at study and outcome level (e.g., risk of bias), and at review level (e.g., incomplete retrieval of identified research, reporting bias). *Comment on the validity of the assumptions, such as transitivity and consistency. Comment on any concerns regarding network geometry (e.g., avoidance of certain comparisons).* | 13-14 |
| Conclusions | 26 | Provide a general interpretation of the results in the context of other evidence, and implications for future research. | 14 |
|  |  |  |  |
| **FUNDING** |  |  |  |
| Funding | 27 | Describe sources of funding for the systematic review and other support (e.g., supply of data); role of funders for the systematic review. This should also include information regarding whether funding has been received from manufacturers of treatments in the network and/or whether some of the authors are content experts with professional conflicts of interest that could affect use of treatments in the network. | 15 |

**Supplementary File S2**

**Table S2-1 Search strategies of MEDLINE (via PubMed)**

| Search number | Query |
| --- | --- |
| 1 | Pleural Effusion[MeSH Terms] |
| 2 | pleura* |
| 3 | effusion* or fluid* |
| 4 | #2 AND #3 |
| 5 | #1 OR #4 |
| 6 | Lung Neoplasms[MeSH Terms] |
| 7 | Bronchopulmonary carcino*[Title/Abstract] OR Cancer of Lung*[Title/Abstract] OR Cancer of the Lung*[Title/Abstract] OR Lung adenocarcimoma*[Title/Abstract] OR Lung Cancer*[Title/Abstract] OR Lung carcinoma*[Title/Abstract] OR Lung malignan*[Title/Abstract] OR Lung Neoplasm*[Title/Abstract] OR Lung Tumo*[Title/Abstract] OR Pulmonary adenocarcinoma*[Title/Abstract] OR Pulmonary Cancer*[Title/Abstract] OR pulmonary carcino*[Title/Abstract] OR pulmonary malignan*[Title/Abstract] OR Pulmonary Neoplasm*[Title/Abstract] OR Pulmonary tumo*[Title/Abstract] |
| 8 | Carcinoma, Non-Small-Cell Lung[MeSH Terms] |
| 9 | Nonsmall Cell Lung Cancer*[Title/Abstract] OR Non Small Cell Lung Cancer*[Title/Abstract] OR Nonsmall Cell Lung Carcinoma*[Title/Abstract] OR Non Small Cell Lung Carcinoma*[Title/Abstract] OR NSCLC[Title/Abstract] |
| 10 | Small Cell Lung Carcinoma[MeSH Terms] |
| 11 | Oat Cell Carcinoma*[Title/Abstract] OR Oat Cell Lung Cancer*[Title/Abstract] OR SCLC[Title/Abstract] OR Small Cell Lung Cancer*[Title/Abstract] OR Small Cell Lung Carcinoma*[Title/Abstract] |
| 12 | #6 OR #7 OR #8 OR #9 OR #10 OR #11 |
| 13 | randomized controlled trial[Publication Type] |
| 14 | controlled clinical trial[Publication Type] |
| 15 | randomized[Title/Abstract] |
| 16 | placebo[Title/Abstract] |
| 17 | drug therapy[MeSH Subheading] |
| 18 | randomly[Title/Abstract] |
| 19 | trial[Title/Abstract] |
| 20 | groups[Title/Abstract] |
| 21 | #13 OR #14 #15 OR #16 OR #17 OR #18 OR #19 OR #20 |
| 22 | #5 AND #12 AND #21 |

**Table S2-2 Search strategies of CENTRAL (THE COCHRANE LIBRARY)**

| Search number | Query |
| --- | --- |
| 1 | MeSH descriptor: [Pleural Effusion] explode all trees |
| 2 | (pleura* near/5 (effusion* or fluid*)):ti,ab,kw(Word variations have been searched) |
| 3 | 1 or 2 |
| 4 | MeSH descriptor: [Lung Neoplasms] explode all trees |
| 5 | Bronchopulmonary carcino* or "Cancer of Lung*" or "Cancer of the Lung*" or "Lung adenocarcimoma*" or "Lung Cancer*" or "Lung carcinoma*" or "Lung malignan*" or "Lung Neoplasm*" or "Lung Tumo*" or "Pulmonary adenocarcinoma*" or "Pulmonary Cancer*" or "pulmonary carcino*" or "pulmonary malignan*" or "Pulmonary Neoplasm*" or "Pulmonary tumo*" |
| 6 | MeSH descriptor: [Carcinoma, Non-Small-Cell Lung] explode all trees |
| 7 | Nonsmall Cell Lung Cancer* or "Non Small Cell Lung Cancer*" or "Nonsmall Cell Lung Carcinoma*" or "Non Small Cell Lung Carcinoma*" or NSCLC |
| 8 | MeSH descriptor: [Small Cell Lung Carcinoma] explode all trees |
| 9 | Oat Cell Carcinoma* or "Oat Cell Lung Cancer*" or SCLC or "Small Cell Lung Cancer*" or "Small Cell Lung Carcinoma*" |
| 10 | 4 or 5 or 6 or 7 or 8 or 9 |
| 11 | 3 and 10 |

**Table S2-3 Search strategies of EMBASE (via OVID)**

| Search number | Query |
| --- | --- |
| 1 | exp Pleural Effusion/ |
| 2 | (pleura* adj5 (effusion* or fluid*)).mp. |
| 3 | 1 or 2 |
| 4 | exp lung tumor/ |
| 5 | ((('bronchopulmonary carcino*':ti,ab or 'cancer') adj3 'lung*') or 'lung adenocarcimoma* ':ti,ab OR'lung cancer*':ti,ab or 'lung carcinoma*':ti,ab or 'lung malignan*':ti,ab or 'lung neoplasm* ':ti,ab or 'lung tumo*':ti,ab or 'pulmonary adenocarcinoma*':ti,ab or 'pulmonary cancer* ':ti,ab or 'pulmonary carcino*':ti,ab or 'pulmonary malignan*':ti,ab or 'pulmonary neoplasm* ':ti,ab or 'pulmonary tumo*':ti,ab).mp. |
| 6 | exp non small cell lung cancer/ or 'nonsmall cell lung cancer*':ti,ab.mp. or 'non small cell lung cancer* ':ti,ab.mp. or 'nonsmall cell lung carcinoma*':ti,ab.mp. or 'non small cell lung carcinoma*':ti,ab.mp. or 'nsclc':ti,ab.mp. |
| 7 | exp small cell lung cancer/ or 'oat cell carcinoma*':ti,ab.mp. or 'oat cell lung cancer*':ti,ab.mp. or 'sclc':ti,ab.mp. or 'small cell lung cancer*':ti,ab.mp. or 'small cell lung carcinoma*':ti,ab.mp. |
| 8 | 4 or 5 or 6 or 7 |
| 9 | random$.tw. |
| 10 | factorial$.tw. |
| 11 | crossover$.tw. |
| 12 | cross over$.tw. |
| 13 | cross-over$.tw. |
| 14 | placebo$.tw. |
| 15 | (doubl$ adj blind$).tw. |
| 16 | (singl$ adj blind$).tw. |
| 17 | assign$.tw. |
| 18 | allocat$.tw. |
| 19 | volunteer$.tw. |
| 20 | Crossover Procedure/ |
| 21 | double-blind procedure.tw. |
| 22 | Randomized Controlled Trial/ |
| 23 | Single Blind Procedure/ |
| 24 | or/9-23 |
| 25 | (animal/ or nonhuman/) not human/ |
| 26 | 24 not 25 |
| 27 | 3 and 8 and 26 |

**S2-4 Search strategies of CNKI**

((TKA = '注射' OR TKA = '注射液' OR TKA = '注射剂' OR TKA = '提取物' OR TKA = '艾迪' OR TKA = '爱迪' OR TKA = '苦参' OR TKA = '岩舒' OR TKA = '华蟾素' OR TKA = '康莱特' OR TKA = '薏苡仁' OR TKA = 'ZCE' OR TKA = '消癌平' OR TKA = '通关藤' OR TKA = '鸦胆子' OR TKA = '安体康' OR TKA = '康艾' OR TKA = '参芪扶正' OR TKA = '黄芪多糖' OR TKA = '猪苓多糖' OR TKA = '猪苓' OR TKA = '黄芪') OR ( TKA = '中西医' OR TKA = '中西医结合' OR TKA = '中医' OR TKA = '中药' OR TKA = '中医药' OR TKA = '中成药' OR TKA = '中药注射液' OR TKA = '中药注射剂')) AND (FT = '灌注' OR FT = '腔内' OR FT = '胸内' OR FT = '胸腔内' OR FT= '注射' OR FT = '注入') AND (SU = '胸水' OR SU = '胸水液' OR SU = '胸腔积液' OR SU = '恶性胸腔积液' OR SU = '恶性胸水' OR SU = '癌性胸水' OR SU = '癌性胸腔积液' OR SU = '癌胸水' OR SU = '癌相关胸水') AND (FT = '肺癌' OR FT = '肺肿瘤' OR FT = '肺恶性肿瘤' OR FT = '非小细胞肺癌' OR FT = '肺腺癌' OR FT = '肺鳞癌' OR FT = '小细胞肺癌' OR FT = 'NSCLC' OR FT = 'SCLC') AND (FT = '随机' OR FT = '对照' OR FT = '随机分配' OR FT = '临床观察' OR FT = '临床研究' OR FT = '临床试验' OR FT = '疗效观察')

**S2-5 Search strategies of WanFang**

主题:(注射 OR 注射液 OR 注射剂 OR 提取物 OR 艾迪 OR 爱迪 OR 苦参 OR 岩舒 OR 华蟾素 OR 康莱特 OR 薏苡仁 OR ZCE OR 消癌平 OR 通关藤 OR 鸦胆子 OR 安体康 OR 康艾 OR 参芪扶正 OR 黄芪多糖 OR 猪苓多糖 OR 猪苓 OR 黄芪 OR 中西医 OR 中西医结合 OR 中医 OR 中药 OR 中医药 OR 中成药 OR 中药注射液 OR 中药注射剂) and 全部:( 灌注 OR 腔内 OR 胸内 OR 胸腔内 OR 注射 OR 注入) and 主题:(胸水 OR 胸水液 OR 胸腔积液 OR 恶性胸腔积液 OR 恶性胸水 OR 癌性胸水 OR 癌性胸腔积液 OR 癌胸水 OR 癌相关胸水) and 全部:(肺癌 OR 肺肿瘤 OR 肺恶性肿瘤 OR 非小细胞肺癌 OR 肺腺癌 OR 肺鳞癌 OR 小细胞肺癌 OR NSCLC OR SCLC) and 全部:(随机 OR 对照 OR 随机分配 OR 临床观察 OR 临床研究 OR 临床试验 OR 疗效观察)

**S2-6 Search strategies of VIP**

(R=(注射 OR 注射液 OR 注射剂 OR 提取物 OR 艾迪 OR 爱迪 OR 苦参 OR 岩舒 OR 华蟾素 OR 康莱特 OR 薏苡仁 OR ZCE OR 消癌平 OR 通关藤 OR 鸦胆子 OR 安体康 OR 康艾 OR 参芪扶正 OR 黄芪多糖 OR 猪苓多糖 OR 猪苓 OR 黄芪) OR R=(中西医 OR 中西医结合 OR 中医 OR 中药 OR 中医药 OR 中成药 OR 中药注射液 OR 中药注射剂)) AND R=(灌注 OR 腔内 OR 胸内 OR 胸腔内 OR 注射 OR 注入) AND R=(胸水 OR 胸水液 OR 胸腔积液 OR 恶性胸腔积液 OR 恶性胸水 OR 癌性胸水 OR 癌性胸腔积液 OR 癌胸水 OR 癌相关胸水) AND U=(肺癌 OR 肺肿瘤 OR 肺恶性肿瘤 OR 非小细胞肺癌 OR 肺腺癌 OR 肺鳞癌 OR 小细胞肺癌 OR NSCLC OR SCLC) AND U=(随机 OR 对照 OR 随机分配 OR 临床观察 OR 临床研究 OR 临床试验 OR 疗效观察)

**S2-7 Search strategies of SinoMed**

1 "胸腔积液, 恶性"[不加权:扩展]

2 "胸水"[常用字段:智能] OR "胸水液"[常用字段:智能] OR "胸腔积液"[常用字段:智能] OR "恶性胸腔积液"[常用字段:智能] OR "恶性胸水"[常用字段:智能] OR "癌性胸水"[常用字段:智能] OR "癌性胸腔积液"[常用字段:智能] OR "癌胸水"[常用字段:智能] OR "癌相关胸水"[常用字段:智能]

3 (#2) OR (#1)

4 "肺肿瘤"[不加权:扩展]

5 "肺癌"[常用字段:智能] OR "肺肿瘤"[常用字段:智能] OR "肺恶性肿瘤"[常用字段:智能] OR "非小细胞肺癌"[常用字段:智能] OR "肺腺癌"[常用字段:智能] OR "肺鳞癌"[常用字段:智能] OR "小细胞肺癌"[常用字段:智能] OR "NSCLC"[常用字段:智能] OR "SCLC"[常用字段:智能]

6 (#5) OR (#4)

7 (#6) AND (#3)

8 "中西医结合"[不加权:扩展]

1. "中西医"[常用字段:智能] OR "中西医结合"[常用字段:智能] OR "中医"[常用字段:智能] OR "中药"[常用字段:智能] OR "中医药"[常用字段:智能] OR "中成药"[常用字段:智能] OR "中药注射液"[常用字段:智能] OR "中药注射剂"[常用字段:智能]
2. "注射剂"[不加权:扩展]
3. "注射"[常用字段:智能] OR "注射液"[常用字段:智能] OR "注射剂"[常用字段:智能] OR "提取物"[常用字段:智能] OR "艾迪"[常用字段:智能] OR "爱迪"[常用字段:智能] OR "苦参"[常用字段:智能] OR "岩舒"[常用字段:智能] OR "华蟾素"[常用字段:智能] OR "康莱特"[常用字段:智能] OR "薏苡仁"[常用字段:智能] OR "ZCE"[常用字段:智能] OR "消癌平"[常用字段:智能] OR "通关藤"[常用字段:智能] OR "鸦胆子"[常用字段:智能] OR "安体康"[常用字段:智能] OR "康艾"[常用字段:智能] OR "参芪扶正"[常用字段:智能] OR "黄芪多糖"[常用字段:智能] OR "猪苓多糖"[常用字段:智能] OR "猪苓"[常用字段:智能] OR "黄芪"[常用字段:智能]
4. (#11) OR (#10) OR (#9) OR (#8)
5. "灌注"[常用字段:智能] OR "腔内"[常用字段:智能] OR "胸内"[常用字段:智能] OR "胸腔内"[常用字段:智能] OR "注射"[常用字段:智能] OR "注入"[常用字段:智能]
6. "随机对照试验"[不加权:扩展]
7. "随机"[常用字段:智能] OR "对照"[常用字段:智能] OR "随机分配"[常用字段:智能] OR "临床观察"[常用字段:智能] OR "临床研究"[常用字段:智能] OR "临床试验"[常用字段:智能] OR "疗效观察"[常用字段:智能]
8. (#15) OR (#14)
9. (#16) AND (#13) AND (#12) AND (#7)

**Supplementary File S3**

**Detailed information on included CHIs**

|  | **Name of injection** | **Manufacturer** | **Composition** | **Indications** | **Execution standards and approval numbers** | **Antitumor mechanism** |
| --- | --- | --- | --- | --- | --- | --- |
| 1 | Aidi injection | Guizhou Yibai Pharmaceutical Co., Ltd. | Cantharidin, ginseng, astragalus, Acanthopanax senticosus; auxiliary material is glycerin (for injection). | Primary liver cancer, lung cancer, rectal cancer, malignant lymphoma, gynecological malignant tumors, etc. | National Food and Drug Administration National Drug Standard: WS_3_-B-3809-99-2002  Y-National Pharmaceutical Standard Z52020236; | In vitro tumor inhibition experiments show that it has direct killing and inhibitory effects on cancer cells, and can enhance the body's non-specific and specific immune function. |
| 2 | Compound kushen injection | Shanxi Zhendong Pharmaceutical Co., Ltd. | *Sophora flavescens* Aiton; *Heterosmliax yunnanensis* Gagnep. | Cancer pain and bleeding | National Food and Drug Administration National Drug Standard: WS_3_-B-2752-97-2014  Y-National Pharmaceutical Standard Z14021231; | Fufang Kushen injection has anti-tumor effects by regulating tumor cell proliferation, inducing tumor cell differentiation and apoptosis, inhibiting tumor cell invasion and metastasis, reducing tumor angiogenesis, and regulating body immunity. |
| 3 | Huachansu  Injection | Anhui China Resources Jinchan Pharmaceutical Co., Ltd. | cininobufosin | Intermediate and advanced tumors. | National Food and Drug Administration National Drug Standard: WS_3_-B-3045-98  Y-National Pharmaceutical Standard Z34020273; | Modern pharmacological published studies have shown that cininobufosin and its active compounds (such as bufalin and cininobufosin) have significant anti-tumor activities, reverse the regulation of multi-drug resistance and immune response. |
| 4 | Kangai injection | Changbaishan Pharmaceutical Co., Ltd. | Astragalus, Ginseng, Matrine | Primary liver cancer, lung cancer, rectal cancer, malignant lymphoma and gynecological malignant tumors; leukopenia and hypoxia caused by various causes; | National Food and Drug Administration National Drug Standard: WS-11222 (ZD-1222) -2002-2012Z  Y-National Pharmaceutical Standard Z20026868; | kangai injection has a variety of pharmacological effects: anti-tumor, reduce adverse reactions caused by chemotherapy, and improve the body's immune function. |
| 5 | Kanglaite injection | Zhejiang Kanglaite Pharmaceutical Co., Ltd. | *Coix lacryma-jobi* L. | Primary non-small cell lung cancer and primary liver cancer with deficiency of both qi and Yin and dampness due to spleen deficiency should not be operated on; combined with radiotherapy and chemotherapy, it has certain enhancement operation; it has certain anti-cachexia and analgesic effect for patients with advanced cancer. | National Food and Drug Administration National Drug Standard: WS_3_-301 (Z-038) -2006 (Z) -2013  Y-National Pharmaceutical Standard Z10970091; | Coixenolide is the main active ingredient of kanglaite injection, which exhibited anticancer and immunomodulatory properties. |
| 6 | Yadanzi  injection | Jiangsu Jiuxu Pharmaceutical Co., Ltd.; | *Brucea javanica* (L.) Merr.v | Lung cancer, lung cancer brain metastases and digestive tract tumors. | National Food and Drug Administration National Drug Standard: YBZ12472004  Y-National Pharmaceutical Standard Z19993152; | Brucea javanica oil emulsion could induce the death of cancer cells through a variety of mechanisms.and exhibited higher activity and a broader anti-tumor spectrum in vitro. |

**Supplementary File S4**

**
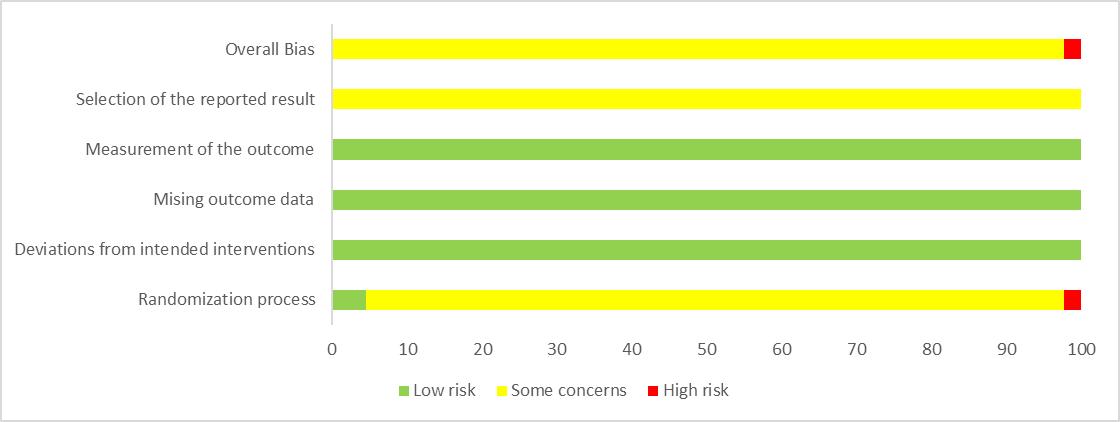
**

**Figure S4** Summary of risk of bias assessment based on revised Cochrane RoB 2 tool.

**Supplementary File S5**

**The forest plot Pairwise Meta-Analysis of six outcomes**

**A
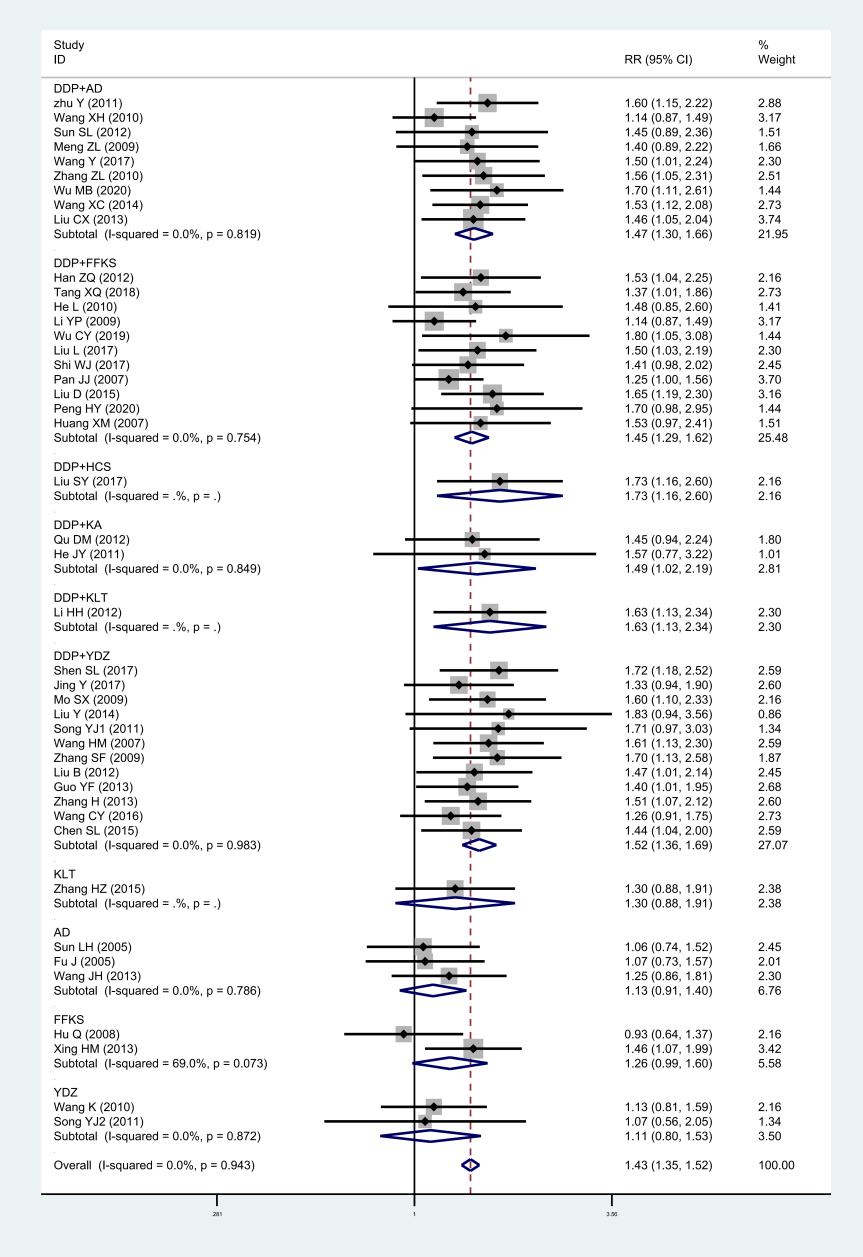
 B
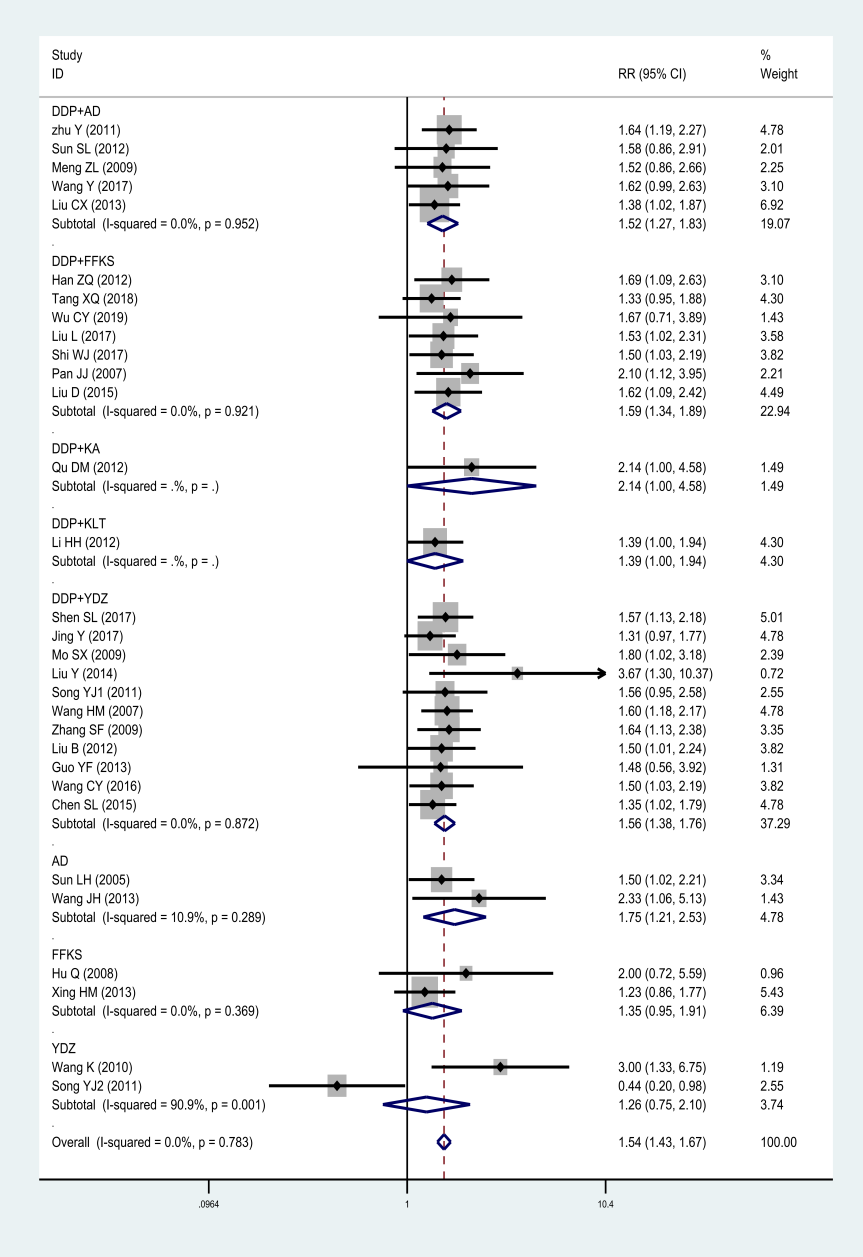
**

**C
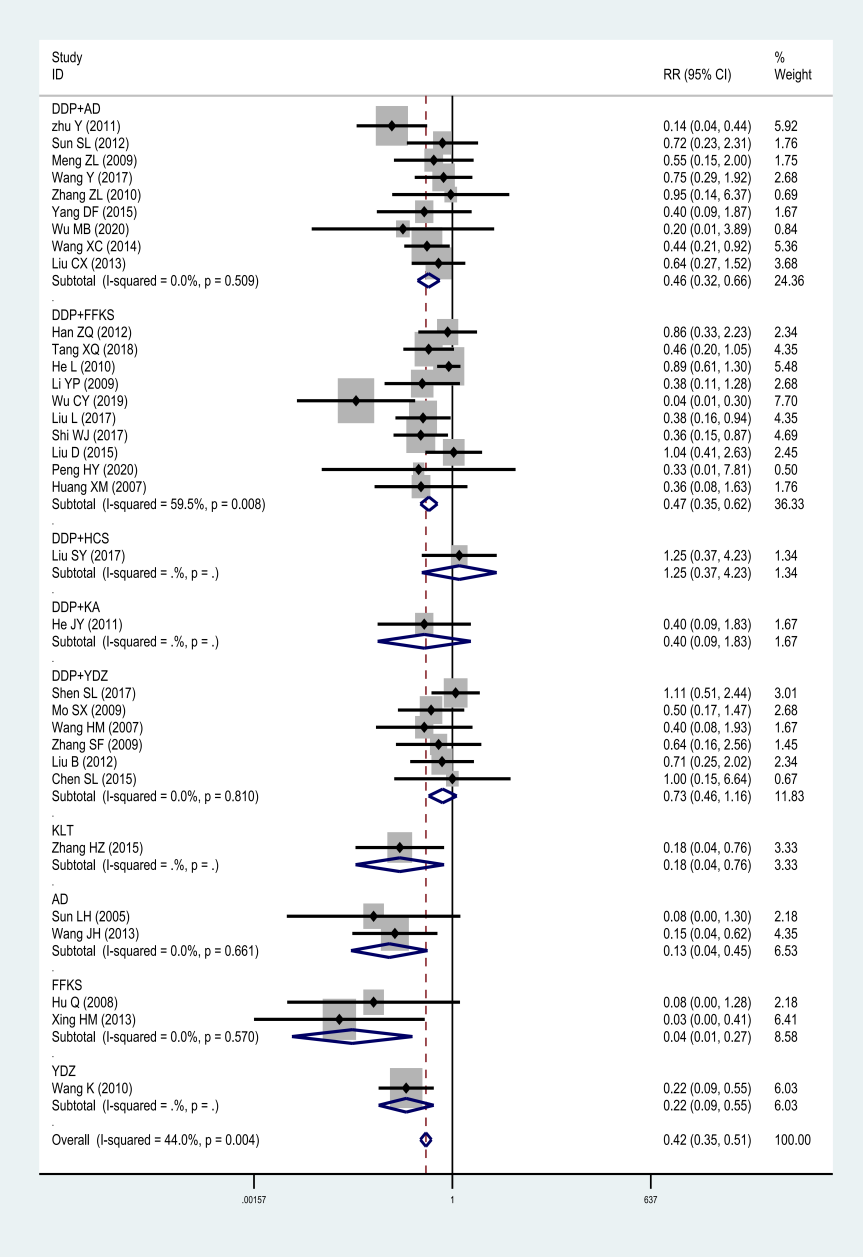
 D
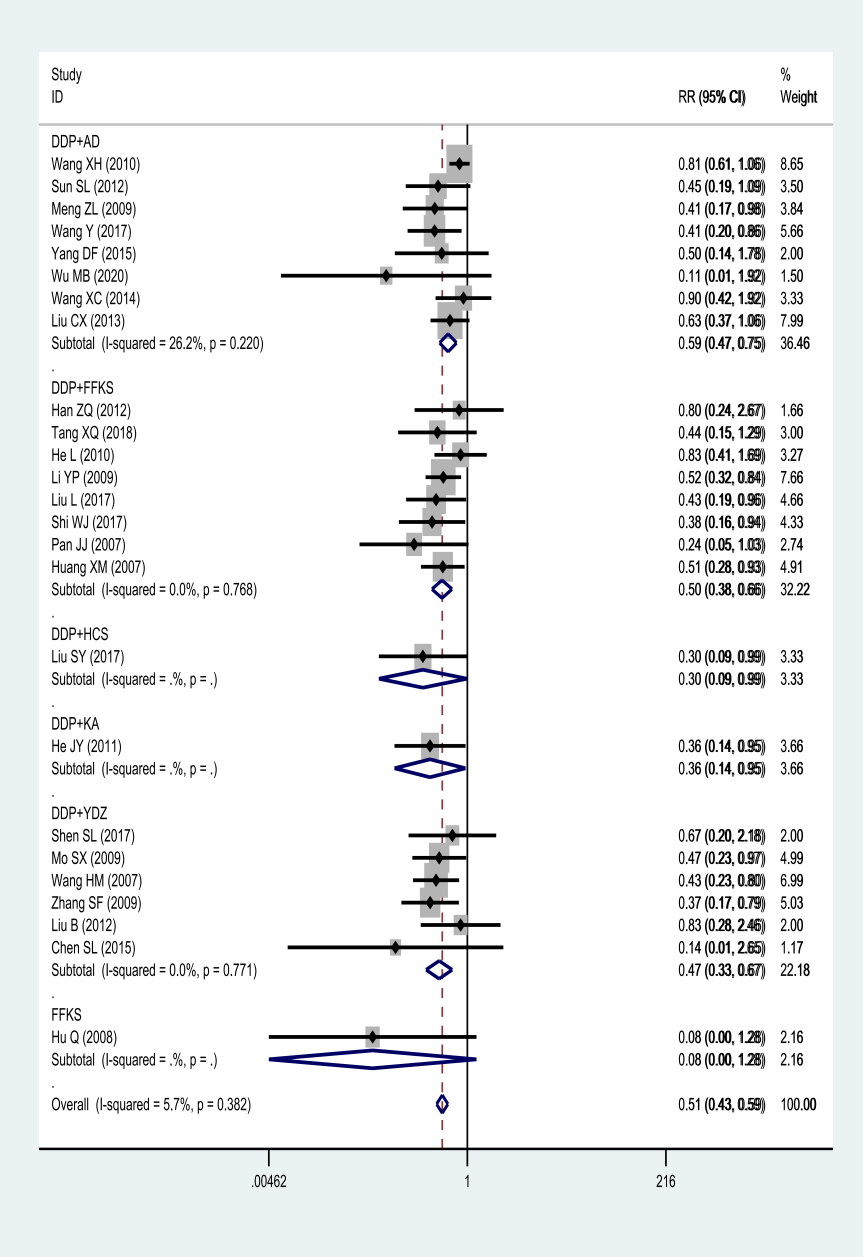
**

**E
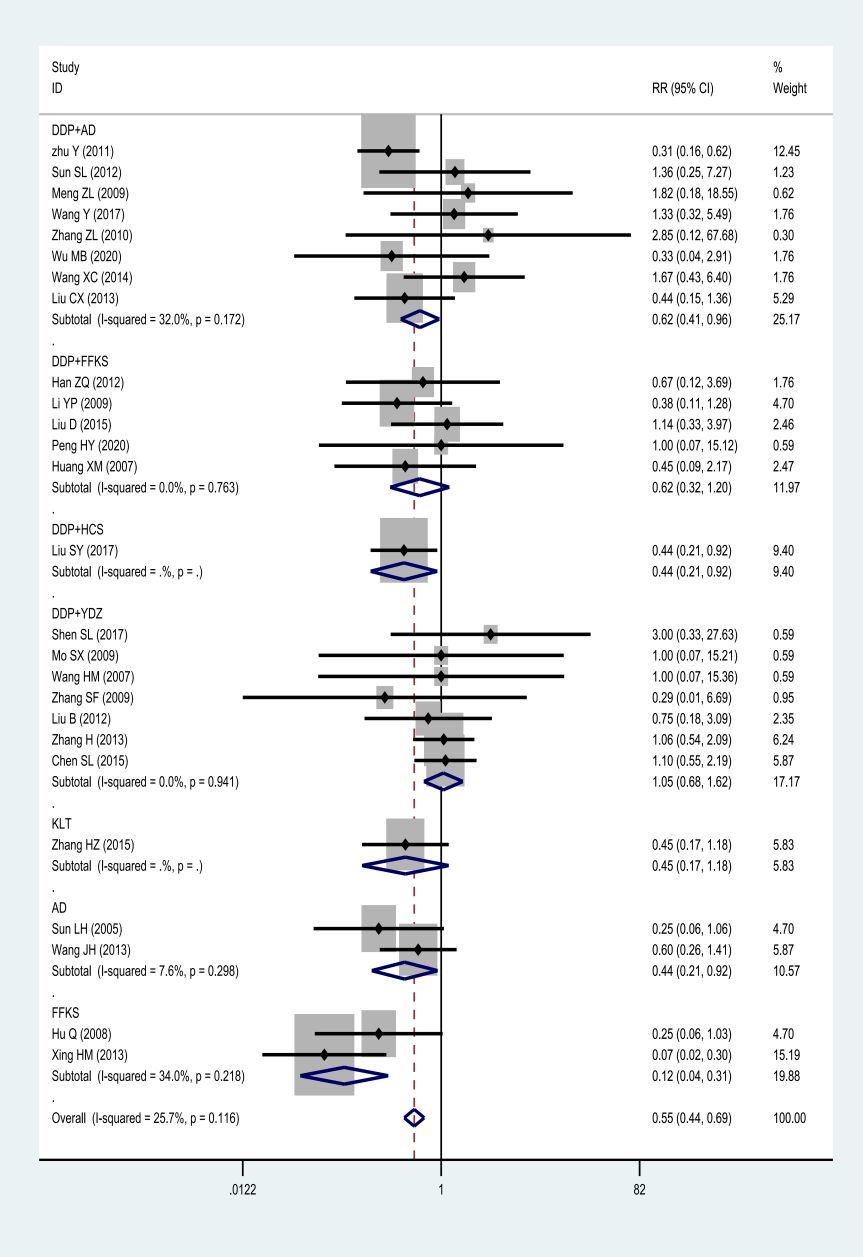
 F
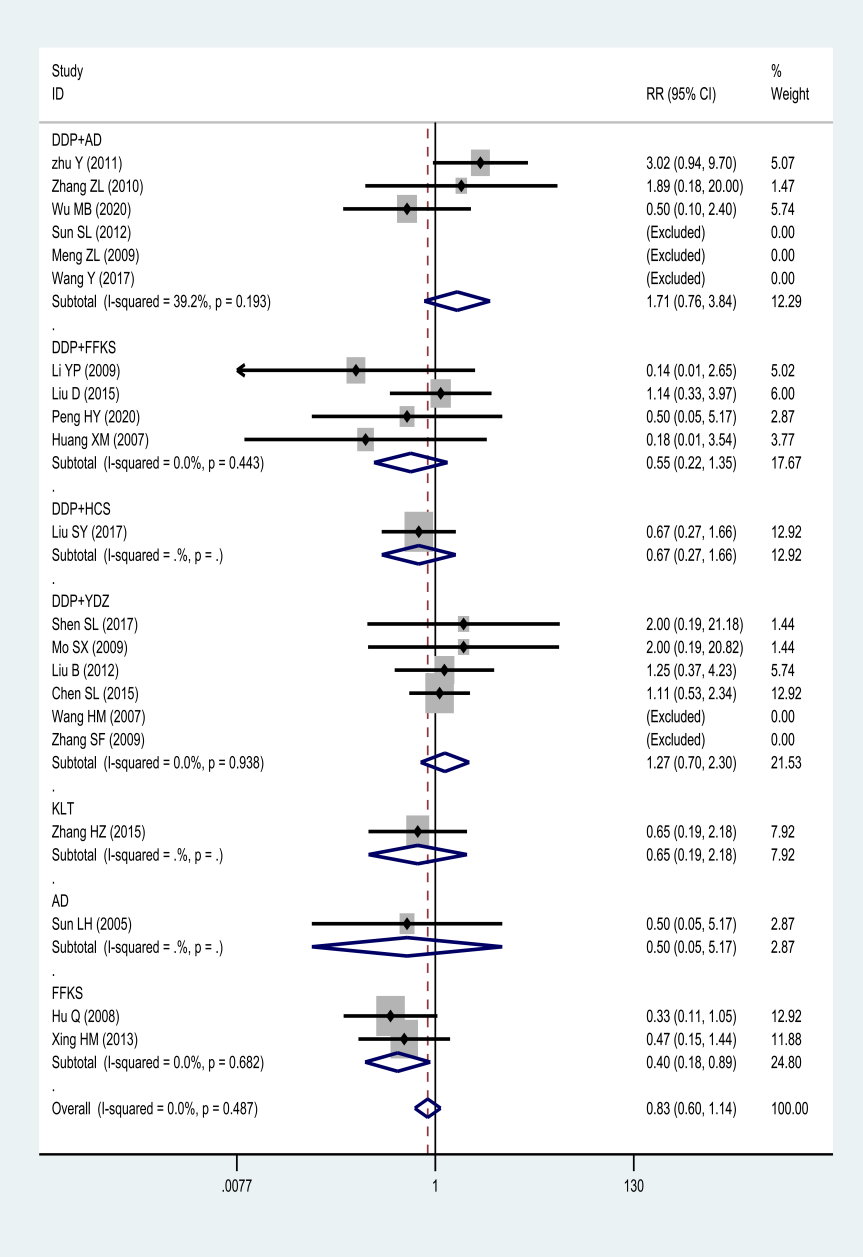
**

**Figure S5** The forest plot Pairwise Meta-Analysis of six outcomes. **(A)** Clinical effective rate; **(B)** The improvement rate of KPS score; **(C)** Incidence of gastrointestinal reactions; **(D)** Incidence of leukopenia; **(E)** Incidence of chest pain; **(F)** Incidence of fever. Note: AD, Aidi injection; DDP, cisplatin; FFKS, Fufang Kushen injection; HCS, Huachansu injection; KA, Kangai injection; KLT, Kanglaite injection; YDZ, Yadanzi injection.

**Supplementary File S6**

# Detailed information of heterogeneity analysis.

**Table S6-1**

| Clinical effective rate | N（studies） | RR 95%CI | *P* | I-squared | *P^,^* |
| --- | --- | --- | --- | --- | --- |
| cisplatin+AD | 9 | 1.47（1.30，1.66） | 0.000 | 0.0% | 0.819 |
| cisplatin+FFKS | 11 | 1.45（1.30，1.62） | 0.000 | 0.0% | 0.754 |
| cisplatin+HCS | 1 | 1.73（1.16，2.60） | 0.008 | .% | . |
| cisplatin+KA | 2 | 1.50（1.02，2.19） | 0.038 | 0.0% | 0.849 |
| cisplatin+KLT | 1 | 1.63（1.13，2.34） | 0.009 | .% | . |
| cisplatin+YDZ | 12 | 1.52（1.37，1.70） | 0.000 | 0.0% | 0.983 |
| KLT | 1 | 1.30（0.88，1.91） | 0.182 | .% | . |
| AD | 3 | 1.13（0.91，1.40） | 0.273 | 0.0% | 0.786 |
| FFKS | 2 | 1.26（0.99，1.60） | 0.064 | 69.0% | **0.073** |
| YDZ | 2 | 1.11（0.80，1.53） | 0.529 | .% | 0.872 |

**Table S6-2**

| The improvement rate of KPS score | N（studies） | RR 95%CI | *P* | I-squared | *P^,^* |
| --- | --- | --- | --- | --- | --- |
| cisplatin+AD | 5 | 1.52（1.27，1.83） | 0.000 | 0.0% | 0.952 |
| cisplatin+FFKS | 7 | 1.59（1.34，1.89） | 0.000 | 0.0% | 0.921 |
| cisplatin+KA | 1 | 2.14（1.00，4.58） | 0.050 | .% | . |
| cisplatin+KLT | 1 | 1.39（1.00，1.94） | 0.053 | .% | . |
| cisplatin+YDZ | 11 | 1.56（1.38，1.76） | 0.000 | 0.0% | 0.872 |
| AD | 2 | 1.75（1.21，2.54） | 0.003 | 10.9% | 0.289 |
| FFKS | 2 | 1.35（0.95，1.91） | 0.095 | 0.0% | 0.369 |
| YDZ | 2 | 1.26（0.75，2.10） | 0.386 | 90.9% | **0.001** |

**Table S6-3**

| Incidence of gastrointestinal reactions | N（studies） | RR 95%CI | *P* | I-squared | *P^,^* |
| --- | --- | --- | --- | --- | --- |
| cisplatin+AD | 9 | 0.46（0.32，0.66） | 0.000 | 0.0% | 0.509 |
| cisplatin+FFKS | 10 | 0.47（0.35，0.62） | 0.000 | 59.5% | **0.008** |
| cisplatin+HCS | 1 | 1.25（0.37，4.23） | 0.720 | .% | . |
| cisplatin+KA | 1 | 0.40（0.09，1.83） | 0.237 | .% | . |
| cisplatin+YDZ | 6 | 0.73（0.46，1.16） | 0.179 | 0.0% | 0.810 |
| KLT | 1 | 0.18（0.04，0.76） | 0.020 | .% | . |
| AD | 2 | 0.13（0.04，0.45） | 0.001 | 0.0% | 0.661 |
| FFKS | 2 | 0.04（0.01，0.27） | 0.001 | 0.0% | 0.570 |
| YDZ | 1 | 0.22（0.09，0.55） | 0.001 | .% | . |

**Table S6-4**

| Incidence of leukopenia | N（studies） | RR 95%CI | *P* | I-squared | *P^,^* |
| --- | --- | --- | --- | --- | --- |
| cisplatin+AD | 8 | 0.60（0.47，0.75） | 0.000 | 26.2% | 0.220 |
| cisplatin+FFKS | 8 | 0.50（0.38，0.66） | 0.000 | 0.0% | 0.768 |
| cisplatin+HCS | 1 | 0.30（0.09，0.99） | 0.048 | .% | . |
| cisplatin+KA | 1 | 0.36（0.14，0.95） | 0.039 | .% | . |
| cisplatin+YDZ | 6 | 0.47（0.33，0.67） | 0.000 | 0.0% | 0.771 |
| FFKS | 1 | 0.08（0.01，1.28） | 0.074 | .% | . |

**Table S6-5**

| Incidence of chest pain | N（studies） | RR 95%CI | *P* | I-squared | *P^,^* |
| --- | --- | --- | --- | --- | --- |
| cisplatin+AD | 8 | 0.63（0.41，0.96） | 0.032 | 32.0% | 0.172 |
| cisplatin+FFKS | 5 | 0.62（0.32，1.20） | 0.156 | 0.0% | 0.763 |
| cisplatin+HCS | 1 | 0.44（0.21，0.92） | 0.029 | .% | . |
| cisplatin+YDZ | 7 | 1.05（0.68，1.62） | 0.824 | 0.0% | 0.941 |
| KLT | 1 | 0.45（0.17，1.18） | 0.105 | .% | . |
| AD | 2 | 0.44（0.21，0.92） | 0.030 | 7.6% | 0.298 |
| FFKS | 2 | 0.12（0.04，0.31） | 0.000 | 34.0% | 0.218 |

**Table S6-6**

| Incidence of fever | N（studies） | RR 95%CI | *P* | I-squared | *P^,^* |
| --- | --- | --- | --- | --- | --- |
| cisplatin+AD | 6 | 1.71（0.76，3.84） | 0.194 | 39.2% | 0.193 |
| cisplatin+FFKS | 4 | 0.55（0.22，1.35） | 0.193 | 0.0% | 0.443 |
| cisplatin+HCS | 1 | 0.67（0.27，1.66） | 0.382 | .% | . |
| cisplatin+YDZ | 6 | 1.27（0.70，2.30） | 0.438 | 0.0% | 0.938 |
| KLT | 1 | 0.65（0.20，2.18） | 0.485 | .% | . |
| AD | 1 | 0.50（0.05，5.17） | 0.561 | .% | . |
| FFKS | 2 | 0.40（0.18，0.89） | 0.024 | 0.0% | 0.682 |

Note: *P^,^* : the statistical difference of heterogeneity.

**The details of subgroup analysis and sensitivity analysis**

**Table S6-7 Subgroup analysis**

| The total DDP dose (mg) | Incidence of gastrointestinal reactions(DDP+FFKS) | |
| --- | --- | --- |
|  | I-squared | *P^,^* |
| <160 | 0.0% | 0.772 |
| 160-200 (include160) | 24.3% | 0.259 |
| ≥200 | 31.8% | 0.231 |

**Table S6-8 Sensitivity analysis**

| Model | RR 95%CI | *P* | I-squared | *P^,^* |
| --- | --- | --- | --- | --- |
| Fixed | 0.47（0.35，0.62） | 0.000 | 59.5% | **0.008** |
| Random | 0.50（0.30，0.81） | 0.005 | 59.5% | **0.008** |

Note: *P^,^*: the statistical difference of heterogeneity.

Fixed model Random model

Note: Figure of sensitivity about incidence of gastrointestinal reactions with fixed effect model.

**Supplementary File S7**

**
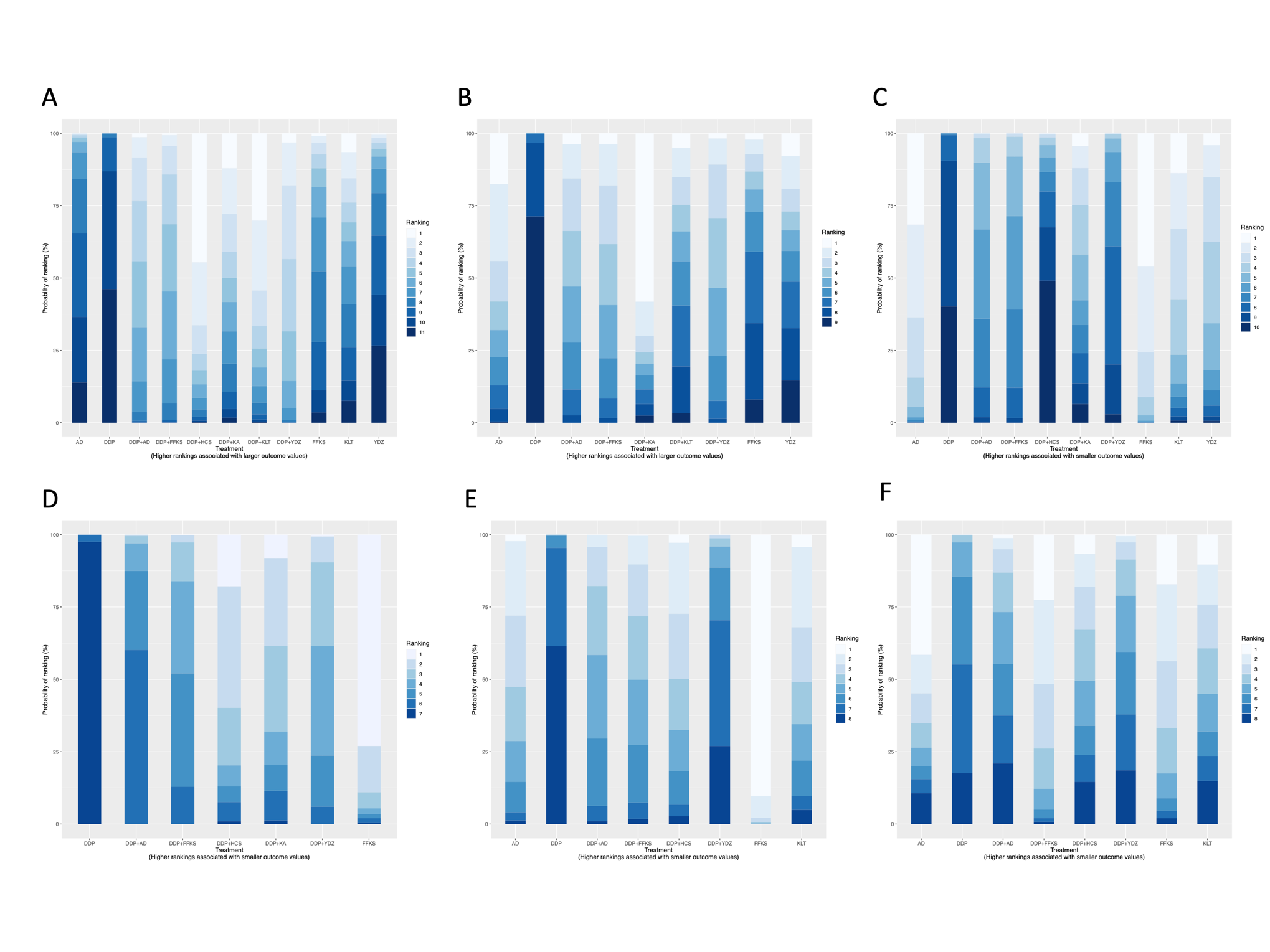
Figure S7** Cumulative ranking plots of different interventions for six outcomes. **(A)** Clinical effective rate; **(B)** The improvement rate of KPS score; **(C)** Incidence of gastrointestinal reactions; **(D)** Incidence of leukopenia; **(E)** Incidence of chest pain; **(F)** Incidence of fever. Note: The size of each colored bar corresponds to the probability of each treatment to be at a specific rank, with the lightest blue ranking the first. AD, Aidi injection; DDP, cisplatin; FFKS, Fufang Kushen injection; HCS, Huachansu injection; KA, Kangai injection; KLT, Kanglaite injection; YDZ, Yadanzi injection.

**Supplementary File S8**


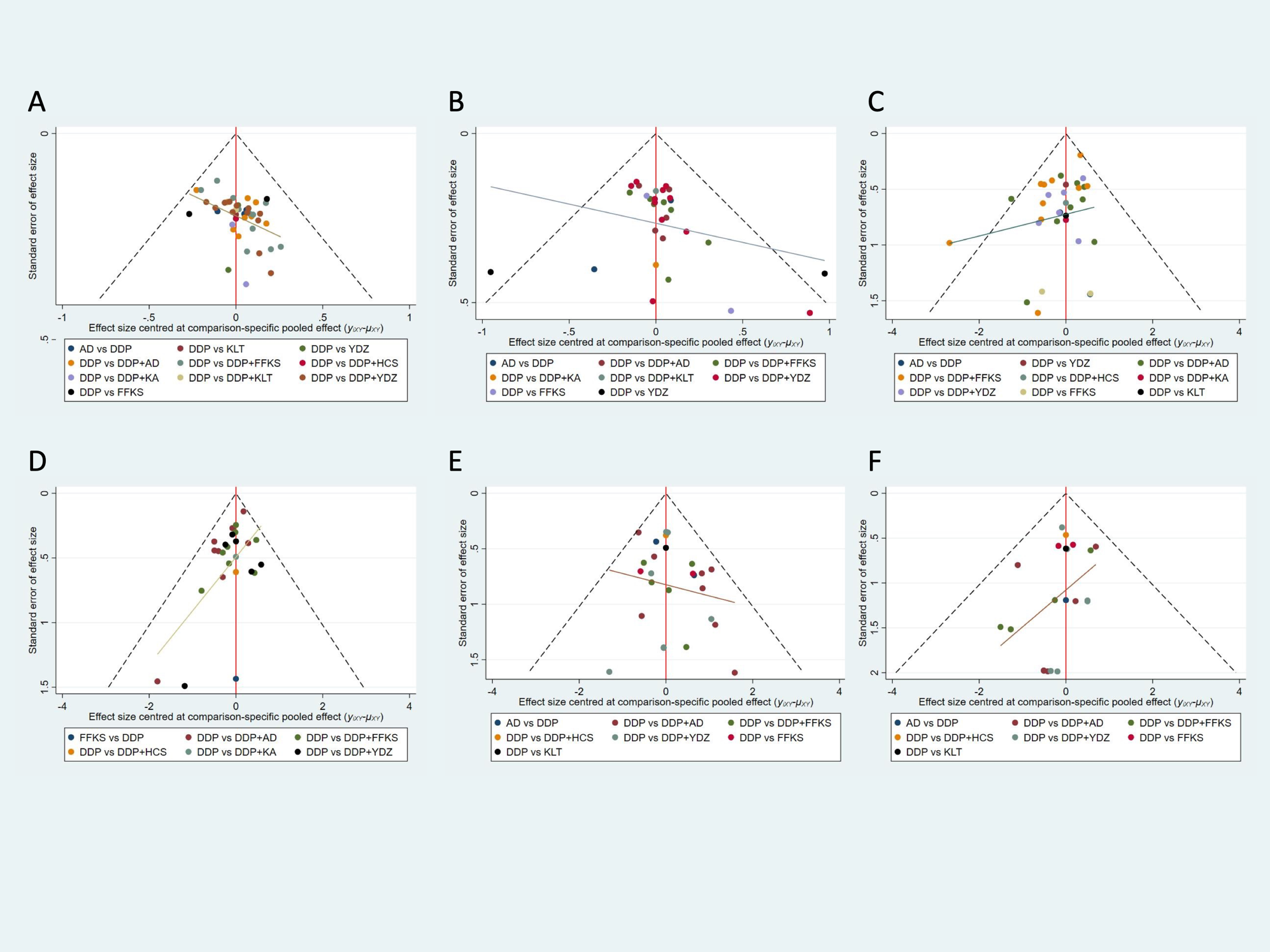
**Figure S8** Comparison-adjusted funnel plot for six outcomes. **(A)** Clinical effective rate; **(B)** The improvement rate of KPS score; **(C)** Incidence of gastrointestinal reactions; **(D)** Incidence of leukopenia; **(E)** Incidence of chest pain; **(F)** Incidence of fever. Note: The comparison-adjusted funnel plot can assess small study effects and potential publication bias involving multiple comparisons, with different color points representing different comparisons. The zero line represents the null hypothesis that the study-specific effect sizes do not differ from the respective comparison-specific pooled effect estimates. AD, Aidi injection; DDP, cisplatin; FFKS, Fufang Kushen injection; HCS, Huachansu injection; KA, Kangai injection; KLT, Kanglaite injection; YDZ, Yadanzi injection.

**Supplementary File S9**

**Table S9-1 CINeMA-Clinical effective rate**

| Comparison | Number of studies | Within-study bias | Reporting bias | Indirectness | Imprecision | Heterogeneity | Incoherence | Confidence rating |
| --- | --- | --- | --- | --- | --- | --- | --- | --- |
| AD:DDP | 3 | Some concerns | Some concerns | No concerns | Some concerns | No concerns | Some concerns | Very low |
| DDP:DDP+AD | 9 | Some concerns | Some concerns | No concerns | No concerns | No concerns | Some concerns | Low |
| DDP:DDP+FFKS | 11 | Some concerns | Some concerns | No concerns | No concerns | No concerns | Some concerns | Low |
| DDP:DDP+HCS | 1 | Some concerns | Some concerns | No concerns | No concerns | No concerns | Some concerns | Low |
| DDP:DDP+KA | 2 | Some concerns | Some concerns | No concerns | No concerns | No concerns | Some concerns | Low |
| DDP:DDP+KLT | 1 | Some concerns | Some concerns | No concerns | No concerns | No concerns | Some concerns | Low |
| DDP:DDP+YDZ | 12 | Some concerns | Some concerns | No concerns | No concerns | No concerns | Some concerns | Low |
| DDP:FFKS | 2 | Some concerns | Some concerns | No concerns | Some concerns | No concerns | Some concerns | Very low |
| DDP:KLT | 1 | Some concerns | Some concerns | No concerns | Some concerns | No concerns | Some concerns | Very low |
| DDP:YDZ | 2 | Some concerns | Some concerns | No concerns | Some concerns | No concerns | Some concerns | Very low |
| AD:DDP+AD | 0 | Some concerns | Some concerns | No concerns | Some concerns | No concerns | Some concerns | Very low |
| AD:DDP+FFKS | 0 | Some concerns | Some concerns | No concerns | Some concerns | No concerns | Some concerns | Very low |
| AD:DDP+HCS | 0 | Some concerns | Some concerns | No concerns | Some concerns | No concerns | Some concerns | Very low |
| AD:DDP+KA | 0 | Some concerns | Some concerns | No concerns | Some concerns | No concerns | Some concerns | Very low |
| AD:DDP+KLT | 0 | Some concerns | Some concerns | No concerns | Some concerns | No concerns | Some concerns | Very low |
| AD:DDP+YDZ | 0 | Some concerns | Some concerns | No concerns | No concerns | No concerns | Some concerns | Very low |
| AD:FFKS | 0 | Some concerns | Some concerns | No concerns | Major concerns | No concerns | Some concerns | Very low |
| AD:KLT | 0 | Some concerns | Some concerns | No concerns | Major concerns | No concerns | Some concerns | Very low |
| AD:YDZ | 0 | Some concerns | Some concerns | No concerns | Major concerns | No concerns | Some concerns | Very low |
| DDP+AD:DDP+FFKS | 0 | Some concerns | Some concerns | No concerns | No concerns | No concerns | Some concerns | Very low |
| DDP+AD:DDP+HCS | 0 | Some concerns | Some concerns | No concerns | Major concerns | No concerns | Some concerns | Very low |
| DDP+AD:DDP+KA | 0 | Some concerns | Some concerns | No concerns | Major concerns | No concerns | Some concerns | Very low |
| DDP+AD:DDP+KLT | 0 | Some concerns | Some concerns | No concerns | Major concerns | No concerns | Some concerns | Very low |
| DDP+AD:DDP+YDZ | 0 | Some concerns | Some concerns | No concerns | No concerns | No concerns | Some concerns | Very low |
| DDP+AD:FFKS | 0 | Some concerns | Some concerns | No concerns | Some concerns | No concerns | Some concerns | Very low |
| DDP+AD:KLT | 0 | Some concerns | Some concerns | No concerns | Major concerns | No concerns | Some concerns | Very low |
| DDP+AD:YDZ | 0 | Some concerns | Some concerns | No concerns | Some concerns | No concerns | Some concerns | Very low |
| DDP+FFKS:DDP+HCS | 0 | Some concerns | Some concerns | No concerns | Some concerns | No concerns | Some concerns | Very low |
| DDP+FFKS:DDP+KA | 0 | Some concerns | Some concerns | No concerns | Major concerns | No concerns | Some concerns | Very low |
| DDP+FFKS:DDP+KLT | 0 | Some concerns | Some concerns | No concerns | Some concerns | Some concerns | Some concerns | Very low |
| DDP+FFKS:DDP+YDZ | 0 | Some concerns | Some concerns | No concerns | Some concerns | No concerns | Some concerns | Very low |
| DDP+FFKS:FFKS | 0 | Some concerns | Some concerns | No concerns | Some concerns | No concerns | Some concerns | Very low |
| DDP+FFKS:KLT | 0 | Some concerns | Some concerns | No concerns | Major concerns | No concerns | Some concerns | Very low |
| DDP+FFKS:YDZ | 0 | Some concerns | Some concerns | No concerns | Some concerns | No concerns | Some concerns | Very low |
| DDP+HCS:DDP+KA | 0 | Some concerns | Some concerns | No concerns | Major concerns | No concerns | Some concerns | Very low |
| DDP+HCS:DDP+KLT | 0 | Some concerns | Some concerns | No concerns | Major concerns | No concerns | Some concerns | Very low |
| DDP+HCS:DDP+YDZ | 0 | Some concerns | Some concerns | No concerns | Major concerns | No concerns | Some concerns | Very low |
| DDP+HCS:FFKS | 0 | Some concerns | Some concerns | No concerns | Some concerns | No concerns | Some concerns | Very low |
| DDP+HCS:KLT | 0 | Some concerns | Some concerns | No concerns | Major concerns | No concerns | Some concerns | Very low |
| DDP+HCS:YDZ | 0 | Some concerns | Some concerns | No concerns | Some concerns | No concerns | Some concerns | Very low |
| DDP+KA:DDP+KLT | 0 | Some concerns | Some concerns | No concerns | Major concerns | No concerns | Some concerns | Very low |
| DDP+KA:DDP+YDZ | 0 | Some concerns | Some concerns | No concerns | Major concerns | No concerns | Some concerns | Very low |
| DDP+KA:FFKS | 0 | Some concerns | Some concerns | No concerns | Major concerns | No concerns | Some concerns | Very low |
| DDP+KA:KLT | 0 | Some concerns | Some concerns | No concerns | Major concerns | No concerns | Some concerns | Very low |
| DDP+KA:YDZ | 0 | Some concerns | Some concerns | No concerns | Some concerns | No concerns | Some concerns | Very low |
| DDP+KLT:DDP+YDZ | 0 | Some concerns | Some concerns | No concerns | Major concerns | No concerns | Some concerns | Very low |
| DDP+KLT:FFKS | 0 | Some concerns | Some concerns | No concerns | Some concerns | No concerns | Some concerns | Very low |
| DDP+KLT:KLT | 0 | Some concerns | Some concerns | No concerns | Major concerns | No concerns | Some concerns | Very low |
| DDP+KLT:YDZ | 0 | Some concerns | Some concerns | No concerns | Some concerns | No concerns | Some concerns | Very low |
| DDP+YDZ:FFKS | 0 | Some concerns | Some concerns | No concerns | Some concerns | No concerns | Some concerns | Very low |
| DDP+YDZ:KLT | 0 | Some concerns | Some concerns | No concerns | Major concerns | No concerns | Some concerns | Very low |
| DDP+YDZ:YDZ | 0 | Some concerns | Some concerns | No concerns | Some concerns | No concerns | Some concerns | Very low |
| FFKS:KLT | 0 | Some concerns | Some concerns | No concerns | Major concerns | No concerns | Some concerns | Very low |
| FFKS:YDZ | 0 | Some concerns | Some concerns | No concerns | Major concerns | No concerns | Some concerns | Very low |
| KLT:YDZ | 0 | Some concerns | Some concerns | No concerns | Major concerns | No concerns | Some concerns | Very low |

**Table S9-2 CINeMA-Improvement rate of KPS score**

| Comparison | Number of studies | Within-study bias | Reporting bias | Indirectness | Imprecision | Heterogeneity | Incoherence | Confidence rating |
| --- | --- | --- | --- | --- | --- | --- | --- | --- |
| AD:DDP | 2 | Some concerns | Some concerns | No concerns | No concerns | No concerns | Some concerns | Low |
| DDP:DDP+AD | 5 | Some concerns | Some concerns | No concerns | No concerns | No concerns | Some concerns | Low |
| DDP:DDP+FFKS | 7 | Some concerns | Some concerns | No concerns | No concerns | No concerns | Some concerns | Low |
| DDP:DDP+KA | 1 | Some concerns | Some concerns | No concerns | Some concerns | No concerns | Some concerns | Very low |
| DDP:DDP+KLT | 1 | Some concerns | Some concerns | No concerns | Some concerns | No concerns | Some concerns | Very low |
| DDP:DDP+YDZ | 11 | Some concerns | Some concerns | No concerns | No concerns | No concerns | Some concerns | Low |
| DDP:FFKS | 2 | Some concerns | Some concerns | No concerns | Some concerns | No concerns | Some concerns | Very low |
| DDP:YDZ | 2 | Some concerns | Some concerns | No concerns | Major concerns | No concerns | Some concerns | Very low |
| AD:DDP+AD | 0 | Some concerns | Some concerns | No concerns | Major concerns | No concerns | Some concerns | Very low |
| AD:DDP+FFKS | 0 | Some concerns | Some concerns | No concerns | Major concerns | No concerns | Some concerns | Very low |
| AD:DDP+KA | 0 | Some concerns | Some concerns | No concerns | Major concerns | No concerns | Some concerns | Very low |
| AD:DDP+KLT | 0 | Some concerns | Some concerns | No concerns | Major concerns | No concerns | Some concerns | Very low |
| AD:DDP+YDZ | 0 | Some concerns | Some concerns | No concerns | Major concerns | No concerns | Some concerns | Very low |
| AD:FFKS | 0 | Some concerns | Some concerns | No concerns | Major concerns | No concerns | Some concerns | Very low |
| AD:YDZ | 0 | Some concerns | Some concerns | No concerns | Major concerns | No concerns | Some concerns | Very low |
| DDP+AD:DDP+FFKS | 0 | Some concerns | Some concerns | No concerns | Major concerns | No concerns | Some concerns | Very low |
| DDP+AD:DDP+KA | 0 | Some concerns | Some concerns | No concerns | Major concerns | No concerns | Some concerns | Very low |
| DDP+AD:DDP+KLT | 0 | Some concerns | Some concerns | No concerns | Major concerns | No concerns | Some concerns | Very low |
| DDP+AD:DDP+YDZ | 0 | Some concerns | Some concerns | No concerns | Some concerns | No concerns | Some concerns | Very low |
| DDP+AD:FFKS | 0 | Some concerns | Some concerns | No concerns | Major concerns | No concerns | Some concerns | Very low |
| DDP+AD:YDZ | 0 | Some concerns | Some concerns | No concerns | Major concerns | No concerns | Some concerns | Very low |
| DDP+FFKS:DDP+KA | 0 | Some concerns | Some concerns | No concerns | Major concerns | No concerns | Some concerns | Very low |
| DDP+FFKS:DDP+KLT | 0 | Some concerns | Some concerns | No concerns | Major concerns | No concerns | Some concerns | Very low |
| DDP+FFKS:DDP+YDZ | 0 | Some concerns | Some concerns | No concerns | Some concerns | No concerns | Some concerns | Very low |
| DDP+FFKS:FFKS | 0 | Some concerns | Some concerns | No concerns | Some concerns | No concerns | Some concerns | Very low |
| DDP+FFKS:YDZ | 0 | Some concerns | Some concerns | No concerns | Major concerns | No concerns | Some concerns | Very low |
| DDP+KA:DDP+KLT | 0 | Some concerns | Some concerns | No concerns | Major concerns | No concerns | Some concerns | Very low |
| DDP+KA:DDP+YDZ | 0 | Some concerns | Some concerns | No concerns | Major concerns | No concerns | Some concerns | Very low |
| DDP+KA:FFKS | 0 | Some concerns | Some concerns | No concerns | Major concerns | No concerns | Some concerns | Very low |
| DDP+KA:YDZ | 0 | Some concerns | Some concerns | No concerns | Major concerns | No concerns | Some concerns | Very low |
| DDP+KLT:DDP+YDZ | 0 | Some concerns | Some concerns | No concerns | Major concerns | No concerns | Some concerns | Very low |
| DDP+KLT:FFKS | 0 | Some concerns | Some concerns | No concerns | Major concerns | No concerns | Some concerns | Very low |
| DDP+KLT:YDZ | 0 | Some concerns | Some concerns | No concerns | Major concerns | No concerns | Some concerns | Very low |
| DDP+YDZ:FFKS | 0 | Some concerns | Some concerns | No concerns | Some concerns | Some concerns | Some concerns | Very low |
| DDP+YDZ:YDZ | 0 | Some concerns | Some concerns | No concerns | Major concerns | No concerns | Some concerns | Very low |
| FFKS:YDZ | 0 | Some concerns | Some concerns | No concerns | Major concerns | No concerns | Some concerns | Very low |

**Table S9-3 CINeMA-Incidence of gastrointestinal reactions**

| Comparison | Number of studies | Within-study bias | Reporting bias | Indirectness | Imprecision | Heterogeneity | Incoherence | Confidence rating |
| --- | --- | --- | --- | --- | --- | --- | --- | --- |
| AD:DDP | 2 | Some concerns | Some concerns | No concerns | No concerns | No concerns | Some concerns | Low |
| DDP:DDP+AD | 9 | Some concerns | Some concerns | No concerns | No concerns | Major concerns | Some concerns | Very low |
| DDP:DDP+FFKS | 10 | Some concerns | Some concerns | No concerns | No concerns | Major concerns | Some concerns | Very low |
| DDP:DDP+HCS | 1 | Some concerns | Some concerns | No concerns | Major concerns | No concerns | Some concerns | Very low |
| DDP:DDP+KA | 1 | Some concerns | Some concerns | No concerns | Major concerns | No concerns | Some concerns | Very low |
| DDP:DDP+YDZ | 6 | Some concerns | Some concerns | No concerns | Major concerns | No concerns | Some concerns | Very low |
| DDP:FFKS | 2 | Some concerns | Some concerns | No concerns | Major concerns | No concerns | Some concerns | Very low |
| DDP:KLT | 1 | Some concerns | Some concerns | No concerns | No concerns | Some concerns | Some concerns | Very low |
| DDP:YDZ | 1 | Some concerns | Some concerns | No concerns | No concerns | No concerns | Some concerns | Low |
| AD:DDP+AD | 0 | Some concerns | Some concerns | No concerns | Some concerns | Some concerns | Some concerns | Very low |
| AD:DDP+FFKS | 0 | Some concerns | Some concerns | No concerns | Some concerns | Some concerns | Some concerns | Very low |
| AD:DDP+HCS | 0 | Some concerns | Some concerns | No concerns | No concerns | Some concerns | Some concerns | Very low |
| AD:DDP+KA | 0 | Some concerns | Some concerns | No concerns | Major concerns | No concerns | Some concerns | Very low |
| AD:DDP+YDZ | 0 | Some concerns | Some concerns | No concerns | No concerns | Some concerns | Some concerns | Very low |
| AD:FFKS | 0 | Some concerns | Some concerns | No concerns | Major concerns | No concerns | Some concerns | Very low |
| AD:KLT | 0 | Some concerns | Some concerns | No concerns | Major concerns | No concerns | Some concerns | Very low |
| AD:YDZ | 0 | Some concerns | Some concerns | No concerns | Major concerns | No concerns | Some concerns | Very low |
| DDP+AD:DDP+FFKS | 0 | Some concerns | Some concerns | No concerns | Major concerns | No concerns | Some concerns | Very low |
| DDP+AD:DDP+HCS | 0 | Some concerns | Some concerns | No concerns | Major concerns | No concerns | Some concerns | Very low |
| DDP+AD:DDP+KA | 0 | Some concerns | Some concerns | No concerns | Major concerns | No concerns | Some concerns | Very low |
| DDP+AD:DDP+YDZ | 0 | Some concerns | Some concerns | No concerns | Major concerns | No concerns | Some concerns | Very low |
| DDP+AD:FFKS | 0 | Some concerns | Some concerns | No concerns | Major concerns | No concerns | Some concerns | Very low |
| DDP+AD:KLT | 0 | Some concerns | Some concerns | No concerns | Major concerns | No concerns | Some concerns | Very low |
| DDP+AD:YDZ | 0 | Some concerns | Some concerns | No concerns | Major concerns | No concerns | Some concerns | Very low |
| DDP+FFKS:DDP+HCS | 0 | Some concerns | Some concerns | No concerns | Major concerns | No concerns | Some concerns | Very low |
| DDP+FFKS:DDP+KA | 0 | Some concerns | Some concerns | No concerns | Major concerns | No concerns | Some concerns | Very low |
| DDP+FFKS:DDP+YDZ | 0 | Some concerns | Some concerns | No concerns | Major concerns | No concerns | Some concerns | Very low |
| DDP+FFKS:FFKS | 0 | Some concerns | Some concerns | No concerns | Major concerns | No concerns | Some concerns | Very low |
| DDP+FFKS:KLT | 0 | Some concerns | Some concerns | No concerns | Major concerns | No concerns | Some concerns | Very low |
| DDP+FFKS:YDZ | 0 | Some concerns | Some concerns | No concerns | Major concerns | No concerns | Some concerns | Very low |
| DDP+HCS:DDP+KA | 0 | Some concerns | Some concerns | No concerns | Major concerns | No concerns | Some concerns | Very low |
| DDP+HCS:DDP+YDZ | 0 | Some concerns | Some concerns | No concerns | Major concerns | No concerns | Some concerns | Very low |
| DDP+HCS:FFKS | 0 | Some concerns | Some concerns | No concerns | Major concerns | No concerns | Some concerns | Very low |
| DDP+HCS:KLT | 0 | Some concerns | Some concerns | No concerns | Major concerns | No concerns | Some concerns | Very low |
| DDP+HCS:YDZ | 0 | Some concerns | Some concerns | No concerns | Some concerns | Some concerns | Some concerns | Very low |
| DDP+KA:DDP+YDZ | 0 | Some concerns | Some concerns | No concerns | Major concerns | No concerns | Some concerns | Very low |
| DDP+KA:FFKS | 0 | Some concerns | Some concerns | No concerns | Major concerns | No concerns | Some concerns | Very low |
| DDP+KA:KLT | 0 | Some concerns | Some concerns | No concerns | Major concerns | No concerns | Some concerns | Very low |
| DDP+KA:YDZ | 0 | Some concerns | Some concerns | No concerns | Major concerns | No concerns | Some concerns | Very low |
| DDP+YDZ:FFKS | 0 | Some concerns | Some concerns | No concerns | Major concerns | No concerns | Some concerns | Very low |
| DDP+YDZ:KLT | 0 | Some concerns | Some concerns | No concerns | Major concerns | No concerns | Some concerns | Very low |
| DDP+YDZ:YDZ | 0 | Some concerns | Some concerns | No concerns | Some concerns | Some concerns | Some concerns | Very low |
| FFKS:KLT | 0 | Some concerns | Some concerns | No concerns | Major concerns | No concerns | Some concerns | Very low |
| FFKS:YDZ | 0 | Some concerns | Some concerns | No concerns | Major concerns | No concerns | Some concerns | Very low |
| KLT:YDZ | 0 | Some concerns | Some concerns | No concerns | Major concerns | No concerns | Some concerns | Very low |

**Table S9-4 CINeMA-Incidence of leukopenia**

| Comparison | Number of studies | Within-study bias | Reporting bias | Indirectness | Imprecision | Heterogeneity | Incoherence | Confidence rating |
| --- | --- | --- | --- | --- | --- | --- | --- | --- |
| DDP:DDP+AD | 8 | Some concerns | Some concerns | No concerns | No concerns | No concerns | Some concerns | Low |
| DDP:DDP+FFKS | 8 | Some concerns | Some concerns | No concerns | No concerns | No concerns | Some concerns | Low |
| DDP:DDP+HCS | 1 | Some concerns | Some concerns | No concerns | No concerns | Some concerns | Some concerns | Very low |
| DDP:DDP+KA | 1 | Some concerns | Some concerns | No concerns | No concerns | Some concerns | Some concerns | Very low |
| DDP:DDP+YDZ | 6 | Some concerns | Some concerns | No concerns | No concerns | No concerns | Some concerns | Low |
| DDP:FFKS | 1 | Some concerns | Some concerns | No concerns | Major concerns | No concerns | Some concerns | Very low |
| DDP+AD:DDP+FFKS | 0 | Some concerns | Some concerns | No concerns | Some concerns | No concerns | Some concerns | Very low |
| DDP+AD:DDP+HCS | 0 | Some concerns | Some concerns | No concerns | Major concerns | No concerns | Some concerns | Very low |
| DDP+AD:DDP+KA | 0 | Some concerns | Some concerns | No concerns | Major concerns | No concerns | Some concerns | Very low |
| DDP+AD:DDP+YDZ | 0 | Some concerns | Some concerns | No concerns | Some concerns | No concerns | Some concerns | Very low |
| DDP+AD:FFKS | 0 | Some concerns | Some concerns | No concerns | No concerns | Some concerns | Some concerns | Very low |
| DDP+FFKS:DDP+HCS | 0 | Some concerns | Some concerns | No concerns | Major concerns | No concerns | Some concerns | Very low |
| DDP+FFKS:DDP+KA | 0 | Some concerns | Some concerns | No concerns | Major concerns | No concerns | Some concerns | Very low |
| DDP+FFKS:DDP+YDZ | 0 | Some concerns | Some concerns | No concerns | Major concerns | No concerns | Some concerns | Very low |
| DDP+FFKS:FFKS | 0 | Some concerns | Some concerns | No concerns | No concerns | No concerns | Some concerns | Very low |
| DDP+HCS:DDP+KA | 0 | Some concerns | Some concerns | No concerns | Major concerns | No concerns | Some concerns | Very low |
| DDP+HCS:DDP+YDZ | 0 | Some concerns | Some concerns | No concerns | Major concerns | No concerns | Some concerns | Very low |
| DDP+HCS:FFKS | 0 | Some concerns | Some concerns | No concerns | No concerns | No concerns | Some concerns | Very low |
| DDP+KA:DDP+YDZ | 0 | Some concerns | Some concerns | No concerns | Major concerns | No concerns | Some concerns | Very low |
| DDP+KA:FFKS | 0 | Some concerns | Some concerns | No concerns | No concerns | No concerns | Some concerns | Very low |
| DDP+YDZ:FFKS | 0 | Some concerns | Some concerns | No concerns | No concerns | No concerns | Some concerns | Very low |

**Table S6-5 CINeMA-Incidence of chest pain**

| Comparison | Number of studies | Within-study bias | Reporting bias | Indirectness | Imprecision | Heterogeneity | Incoherence | Confidence rating |
| --- | --- | --- | --- | --- | --- | --- | --- | --- |
| AD:DDP | 2 | Some concerns | Some concerns | No concerns | Major concerns | No concerns | Some concerns | Very low |
| DDP:DDP+AD | 8 | Some concerns | Some concerns | No concerns | Major concerns | No concerns | Some concerns | Very low |
| DDP:DDP+FFKS | 5 | Some concerns | Some concerns | No concerns | Major concerns | No concerns | Some concerns | Very low |
| DDP:DDP+HCS | 1 | Some concerns | Some concerns | No concerns | Major concerns | No concerns | Some concerns | Very low |
| DDP:DDP+YDZ | 7 | Some concerns | Some concerns | No concerns | Major concerns | No concerns | Some concerns | Very low |
| DDP:FFKS | 2 | Some concerns | Some concerns | No concerns | Major concerns | No concerns | Some concerns | Very low |
| DDP:KLT | 1 | Some concerns | Some concerns | No concerns | Major concerns | No concerns | Some concerns | Very low |
| AD:DDP+AD | 0 | Some concerns | Some concerns | No concerns | Major concerns | No concerns | Some concerns | Very low |
| AD:DDP+FFKS | 0 | Some concerns | Some concerns | No concerns | Major concerns | No concerns | Some concerns | Very low |
| AD:DDP+HCS | 0 | Some concerns | Some concerns | No concerns | Major concerns | No concerns | Some concerns | Very low |
| AD:DDP+YDZ | 0 | Some concerns | Some concerns | No concerns | Major concerns | No concerns | Some concerns | Very low |
| AD:FFKS | 0 | Some concerns | Some concerns | No concerns | Major concerns | No concerns | Some concerns | Very low |
| AD:KLT | 0 | Some concerns | Some concerns | No concerns | Major concerns | No concerns | Some concerns | Very low |
| DDP+AD:DDP+FFKS | 0 | Some concerns | Some concerns | No concerns | Major concerns | No concerns | Some concerns | Very low |
| DDP+AD:DDP+HCS | 0 | Some concerns | Some concerns | No concerns | Major concerns | No concerns | Some concerns | Very low |
| DDP+AD:DDP+YDZ | 0 | Some concerns | Some concerns | No concerns | Major concerns | No concerns | Some concerns | Very low |
| DDP+AD:FFKS | 0 | Some concerns | Some concerns | No concerns | Major concerns | No concerns | Some concerns | Very low |
| DDP+AD:KLT | 0 | Some concerns | Some concerns | No concerns | Major concerns | No concerns | Some concerns | Very low |
| DDP+FFKS:DDP+HCS | 0 | Some concerns | Some concerns | No concerns | Major concerns | No concerns | Some concerns | Very low |
| DDP+FFKS:DDP+YDZ | 0 | Some concerns | Some concerns | No concerns | Major concerns | No concerns | Some concerns | Very low |
| DDP+FFKS:FFKS | 0 | Some concerns | Some concerns | No concerns | Major concerns | No concerns | Some concerns | Very low |
| DDP+FFKS:KLT | 0 | Some concerns | Some concerns | No concerns | Major concerns | No concerns | Some concerns | Very low |
| DDP+HCS:DDP+YDZ | 0 | Some concerns | Some concerns | No concerns | Major concerns | No concerns | Some concerns | Very low |
| DDP+HCS:FFKS | 0 | Some concerns | Some concerns | No concerns | Major concerns | No concerns | Some concerns | Very low |
| DDP+HCS:KLT | 0 | Some concerns | Some concerns | No concerns | Major concerns | No concerns | Some concerns | Very low |
| DDP+YDZ:FFKS | 0 | Some concerns | Some concerns | No concerns | Major concerns | No concerns | Some concerns | Very low |
| DDP+YDZ:KLT | 0 | Some concerns | Some concerns | No concerns | Major concerns | No concerns | Some concerns | Very low |
| FFKS:KLT | 0 | Some concerns | Some concerns | No concerns | Major concerns | No concerns | Some concerns | Very low |

**Table S9-6 CINeMA-Incidence of fever**

| Comparison | Number of studies | Within-study bias | Reporting bias | Indirectness | Imprecision | Heterogeneity | Incoherence | Confidence rating |
| --- | --- | --- | --- | --- | --- | --- | --- | --- |
| AD:DDP | 1 | Some concerns | Some concerns | No concerns | Major concerns | No concerns | Some concerns | Very low |
| DDP:DDP+AD | 6 | Some concerns | Some concerns | No concerns | Major concerns | No concerns | Some concerns | Very low |
| DDP:DDP+FFKS | 4 | Some concerns | Some concerns | No concerns | Major concerns | No concerns | Some concerns | Very low |
| DDP:DDP+HCS | 1 | Some concerns | Some concerns | No concerns | Major concerns | No concerns | Some concerns | Very low |
| DDP:DDP+YDZ | 6 | Some concerns | Some concerns | No concerns | Major concerns | No concerns | Some concerns | Very low |
| DDP:FFKS | 2 | Some concerns | Some concerns | No concerns | Major concerns | No concerns | Some concerns | Very low |
| DDP:KLT | 1 | Some concerns | Some concerns | No concerns | Major concerns | No concerns | Some concerns | Very low |
| AD:DDP+AD | 0 | Some concerns | Some concerns | No concerns | Major concerns | No concerns | Some concerns | Very low |
| AD:DDP+FFKS | 0 | Some concerns | Some concerns | No concerns | Major concerns | No concerns | Some concerns | Very low |
| AD:DDP+HCS | 0 | Some concerns | Some concerns | No concerns | Major concerns | No concerns | Some concerns | Very low |
| AD:DDP+YDZ | 0 | Some concerns | Some concerns | No concerns | Major concerns | No concerns | Some concerns | Very low |
| AD:FFKS | 0 | Some concerns | Some concerns | No concerns | Major concerns | No concerns | Some concerns | Very low |
| AD:KLT | 0 | Some concerns | Some concerns | No concerns | Major concerns | No concerns | Some concerns | Very low |
| DDP+AD:DDP+FFKS | 0 | Some concerns | Some concerns | No concerns | Major concerns | No concerns | Some concerns | Very low |
| DDP+AD:DDP+HCS | 0 | Some concerns | Some concerns | No concerns | Major concerns | No concerns | Some concerns | Very low |
| DDP+AD:DDP+YDZ | 0 | Some concerns | Some concerns | No concerns | Major concerns | No concerns | Some concerns | Very low |
| DDP+AD:FFKS | 0 | Some concerns | Some concerns | No concerns | Major concerns | No concerns | Some concerns | Very low |
| DDP+AD:KLT | 0 | Some concerns | Some concerns | No concerns | Major concerns | No concerns | Some concerns | Very low |
| DDP+FFKS:DDP+HCS | 0 | Some concerns | Some concerns | No concerns | Major concerns | No concerns | Some concerns | Very low |
| DDP+FFKS:DDP+YDZ | 0 | Some concerns | Some concerns | No concerns | Major concerns | No concerns | Some concerns | Very low |
| DDP+FFKS:FFKS | 0 | Some concerns | Some concerns | No concerns | Major concerns | No concerns | Some concerns | Very low |
| DDP+FFKS:KLT | 0 | Some concerns | Some concerns | No concerns | Major concerns | No concerns | Some concerns | Very low |
| DDP+HCS:DDP+YDZ | 0 | Some concerns | Some concerns | No concerns | Major concerns | No concerns | Some concerns | Very low |
| DDP+HCS:FFKS | 0 | Some concerns | Some concerns | No concerns | Major concerns | No concerns | Some concerns | Very low |
| DDP+HCS:KLT | 0 | Some concerns | Some concerns | No concerns | Major concerns | No concerns | Some concerns | Very low |
| DDP+YDZ:FFKS | 0 | Some concerns | Some concerns | No concerns | Major concerns | No concerns | Some concerns | Very low |
| DDP+YDZ:KLT | 0 | Some concerns | Some concerns | No concerns | Major concerns | No concerns | Some concerns | Very low |
| FFKS:KLT | 0 | Some concerns | Some concerns | No concerns | Major concerns | No concerns | Some concerns | Very low |
