# Supplementary material for: Chinese herbal injections versus intrapleural cisplatin for lung cancer patients with malignant pleural effusion: A Bayesian network meta-analysis of randomized controlled trials
Source: Front Oncol. 2022 Sep 20;12:942941. doi: 10.3389/fonc.2022.942941 (PMC9531116; doi:10.3389/fonc.2022.942941)
Supplement: Supplementary file 3 [file DataSheet_3.docx]

rm(list=ls())

library(rjags)

library(BUGSnet)

#总有效率

library(readxl)

data.effective <- read_excel("徐老师网状/数据/总有效率.xlsx")

data.slr.effective <- data.prep(arm.data=data.effective,varname.t = "t",varname.s = "study")

View(data.slr)

nma.effective <- nma.model(data=data.slr.effective,outcome = "r",

N="n",type="consistency",family = "binomial",

link = "log",

effects = "random",reference = "DDP")

set.seed(123)

nma.effective.results <- nma.run(nma.effective,n.burnin = 50000,n.iter = 250000,n.chains = 4,thin=1)

nma.diag(nma.effective.results,trace = T,gelman.rubin = T,thin=1,ncol=2,nrow=2,plot_prompt = F)

nma.effective.league <- nma.league(nma.effective.results,digits=2)

write.csv(nma.effective.league$table,"effective_league.csv")

nma.effective.rank <- nma.rank(nma.effective.results,largerbetter = T)

nma.effective.rank$sucraplot

nma.effective.rank$rankogram

write.csv(nma.effective.rank$ranktable,"徐老师网状/结果/effective_rank.csv")

#kps

data.kps <- read_excel("徐老师网状/数据/kps.xlsx")

data.slr.kps <- data.prep(arm.data=data.kps,varname.t = "t",varname.s = "study")

View(data.slr)

nma.kps <- nma.model(data=data.slr.kps,outcome = "r",

N="n",type="consistency",family = "binomial",

link = "log",

effects = "random",reference = "DDP")

set.seed(123)

nma.kps.results <- nma.run(nma.kps,n.burnin = 50000,n.iter = 200000,n.chains = 4,thin=1)

nma.diag(nma.kps.results,trace = T,gelman.rubin = T,thin=1,ncol=2,nrow=2,plot_prompt = F)

nma.kps.league <- nma.league(nma.kps.results,digits=2)

write.csv(nma.kps.league$table,"kps_league.csv")

nma.kps.rank <- nma.rank(nma.kps.results,largerbetter = T)

nma.kps.rank$sucraplot

nma.kps.rank$rankogram

write.csv(nma.kps.rank$ranktable,"徐老师网状/结果/kps_rank.csv")

#胃肠道反应

library(readxl)

data.gastric <- read_excel("徐老师网状/数据/gastric.xlsx")

data.slr.gastric <- data.prep(arm.data=data.gastric,varname.t = "t",varname.s = "study")

nma.gastric <- nma.model(data=data.slr.gastric,outcome = "r",

N="n",type="consistency",family = "binomial",

link = "log",

effects = "random",reference = "DDP")

set.seed(123)

nma.gastric.results <- nma.run(nma.gastric,n.burnin = 50000,n.iter = 250000,n.chains = 4,thin=1)

nma.diag(nma.gastric.results,trace = T,gelman.rubin = T,thin=1,ncol=2,nrow=2,plot_prompt = F)

nma.gastric.league <- nma.league(nma.gastric.results,digits=2)

print(nma.gastric.league)

write.csv(nma.gastric.league$table,"徐老师网状/结果/gastric_league.csv")

nma.gastric.rank <- nma.rank(nma.gastric.results,largerbetter = F)

nma.gastric.rank$sucraplot

nma.gastric.rank$rankogram

nma.gastric.rank$ranktable

nma.gastric.rank$sucratable

write.csv(nma.gastric.rank$ranktable,"徐老师网状/结果/gastric_rank.csv")

write.csv(nma.gastric.rank$sucratable,"徐老师网状/结果/gastric_sucra.csv")

#白细胞减少

data.wbc <- read_excel("徐老师网状/数据/wbc.xlsx")

data.slr.wbc <- data.prep(arm.data=data.wbc,varname.t = "t",varname.s = "study")

nma.wbc <- nma.model(data=data.slr.wbc,outcome = "r",

N="n",type="consistency",family = "binomial",

link = "log",

effects = "random",reference = "DDP")

set.seed(123)

nma.wbc.results <- nma.run(nma.wbc,n.burnin = 50000,n.iter = 250000,n.chains = 4,thin=1)

nma.diag(nma.wbc.results,trace = T,gelman.rubin = T,thin=1,ncol=2,nrow=2,plot_prompt = F)

nma.wbc.league <- nma.league(nma.wbc.results,digits=2)

write.csv(nma.wbc.league$table,"徐老师网状/结果/wbc_league.csv")

nma.wbc.rank <- nma.rank(nma.wbc.results,largerbetter = F)

nma.wbc.rank$sucraplot

nma.wbc.rank$rankogram

write.csv(nma.wbc.rank$ranktable,"徐老师网状/结果/wbc_rank.csv")

#胸痛

data.chestpain <- read_excel("徐老师网状/数据/chestpain.xlsx")

data.slr.chestpain <- data.prep(arm.data=data.chestpain,varname.t = "t",varname.s = "study")

nma.chestpain <- nma.model(data=data.slr.chestpain,outcome = "r",

N="n",type="consistency",family = "binomial",

link = "log",

effects = "random",reference = "DDP")

set.seed(123)

nma.chestpain.results <- nma.run(nma.chestpain,n.burnin = 50000,n.iter = 250000,n.chains = 4,thin=1)

nma.diag(nma.chestpain.results,trace = T,gelman.rubin = T,thin=1,ncol=2,nrow=2,plot_prompt = F)

nma.chestpain.league <- nma.league(nma.chestpain.results,digits=2)

write.csv(nma.chestpain.league$table,"徐老师网状/结果/chestpain_league.csv")

nma.chestpain.rank <- nma.rank(nma.chestpain.results,largerbetter = F)

nma.chestpain.rank$sucraplot

nma.chestpain.rank$rankogram

write.csv(nma.chestpain.rank$ranktable,"徐老师网状/结果/chestpain_rank.csv")

#发热

data.fever <- read_excel("徐老师网状/数据/fever.xlsx")

data.slr.fever <- data.prep(arm.data=data.fever,varname.t = "t",varname.s = "study")

nma.fever <- nma.model(data=data.slr.fever,outcome = "r",

N="n",type="consistency",family = "binomial",

link = "log",

effects = "random",reference = "DDP")

set.seed(123)

nma.fever.results <- nma.run(nma.fever,n.burnin = 50000,n.iter = 250000,n.chains = 4,thin=1)

nma.diag(nma.fever.results,trace = T,gelman.rubin = T,thin=1,ncol=2,nrow=2,plot_prompt = F)

nma.fever.league <- nma.league(nma.fever.results,digits=2)

write.csv(nma.fever.league$table,"徐老师网状/结果/fever_league.csv")

nma.fever.rank <- nma.rank(nma.fever.results,largerbetter = F)

nma.fever.rank$sucraplot

nma.fever.rank$rankogram

write.csv(nma.fever.rank$ranktable,"徐老师网状/结果/fever_rank.csv")
